# Depletion of PSMD14 suppresses bladder cancer proliferation by regulating GPX4

**DOI:** 10.7717/peerj.14654

**Published:** 2023-01-06

**Authors:** Changxin Jia, Xin Zhang, Tingting Qu, Xiuyun Wu, Yu Li, Yang Zhao, Lijiang Sun, Qing Wang

**Affiliations:** 1 Department of Anesthesiology, The Affiliated Hospital of Qingdao University, Qingdao, Shandong, China; 2 Department of Pathology, The Affiliated Hospital of Qingdao University, Qingdao, Shandong, China; 3 Department of Urology Surgery, The Affiliated Hospital of Qingdao University, Qingdao, Shandong, China; 4 Department of Endocrine and metabolic diseases, The Affiliated Hospital of Qingdao University, Qingdao, Shandong, China

**Keywords:** PSMD14, GPX4, Bladder cancer

## Abstract

**Objective:**

The aim of this study was to investigate the role of deubiquitinase (DUB) 26S proteasome non-ATPase regulatory subunit 14 (PSMD14) in patients with bladder cancer.

**Methods:**

From 2016 to 2018, 181 patients diagnosed with primary bladder cancer at the Affiliated Hospital of Qingdao University were recruited. The expression of PSMD14 in bladder cancer tissues was tested by immunochemistry. The association between PSMD14 expression and clinical and pathological data and outcomes of bladder cancer patients was determined. Overexpression and knockdown cells were constructed to evaluate the effects of PSMD14 on proliferation of bladder cancer cells.

**Results:**

Our results showed that PSMD14 was significantly overexpressed in bladder cancer tissues compared to adjacent non-tumor tissues (76.24% *vs* 23.76%, *P* = 0.02). The expression of PSMD14 was significantly higher in patients with larger tumor diameters (85.14% *vs* 70.09%, *P* = 0.019) and patients with a family history of cancer (92.16% *vs* 70.00%, *P* = 0.002). Patients with high expression of PSMD14 had poor disease-free survival (DFS) (HR = 2.89, 95% CI [1.247–6.711], *P* = 0.013). Gain and loss of function experiments demonstrated that PSMD14 deficiency inhibited bladder cancer cell proliferation. Additionally, depletion of PSMD14 suppressed bladder cancer cell growth *via* down-regulation of GPX4, and the promotion of PSMD14-induced cell growth was observably reversed by the GPX4 inhibitor RSL3.

**Conclusion:**

We determined that PSMD14 is highly expressed in bladder cancer tissues, and that PSMD14 expression correlated with poor disease-free survival. Depletion of PSMD14 could inhibit the proliferation of bladder cancer cells through the downregulation of GPX4. Therefore, PSMD14 may be an effective target for the treatment of bladder cancer.

## Introduction

Bladder cancer (BC) is one of the most common malignant tumors of the urinary system and causes almost 210,000 annual deaths worldwide (Sung et al., 2021). Therapeutic strategies based on surgical resection in combination with chemo-radiotherapy and immunotherapy have achieved great success in the treatment of this disease. However, the survival status of advanced stage BC is still very poor (Bednova & Leyton, 2020; Ng et al., 2021). Furthermore, most patients experience serious side effects from chemotherapy drugs. Therefore, a better understanding of the molecular mechanisms underlying the progression of BC and the detection of new therapeutic targets is urgently needed.

Deubiquitinase (DUB) 26S proteasome non-ATPase regulatory subunit 14 (PSMD14), also known as RPN11 or POH1, is a protein consisting of 310 amino acids encoded by a 12-exon gene located on chromosome 2q24.2 (Spataro & Buetti-Dinh, 2022). A member of the DUB JAMM domain metalloprotease family, PSMD14 has been shown to participate in various biological processes, such as cellular proliferation, transcriptional regulation, and protein stability (Gallery et al., 2007; Schwarz et al., 2010). PSMD14 is considered an enzyme that can cleave unnecessary proteins by binding and activating the 20S proteasome to form 26S proteasomes (Spataro & Buetti-Dinh, 2022). PSMD14 can reduce DNA–ubiquitin conjugates and maintain embryonic stem cell pluripotency and self-renewal abilities (Buckley et al., 2012). Importantly, PSMD14 is also involved in several types of tumors and is considered a potential therapeutic target for cancer (Wang et al., 2018; Zhi et al., 2019). The expression of PSMD14 is significantly higher in hepatocellular carcinoma than in normal liver tissues, and the knockdown of PSMD14 expression inhibited hepatocellular cancer proliferation and metastasis by down-regulation of GRB2 (Lv et al., 2020). Wang et al. (2018) demonstrated that PSMD14 depletion increased p53 stability, resulting in apoptosis of colorectal cancer (CRC) cells. In esophageal squamous cell carcinoma, knockdown of PSMD14 significantly blocks SNAIL-induced epithelial mesenchymal transition (EMT), thus suppressing tumor cell migration and metastasis (Zhu et al., 2018). Therefore, PSMD14 could be a promising anticancer target used to inhibit tumor growth and block tumor metastasis. However, the effects of PSMD14 on BC progression remain unclear.

Ferroptosis is a novel form of programmed cell death characterized by an iron-dependent accumulation of lipid peroxidation and mitochondrial shrinkage (Liu et al., 2021). Ferroptosis has been demonstrated in different human diseases, including ischemic organ damage and cancer (Jiang, Stockwell & Conrad, 2021). Glutathione peroxidase 4 (GPX4) is a central regulatory marker for ferroptosis that can convert glutathione (GSH) to oxidized glutathione (GSSG) while also reducing lipid hydroperoxides (Zhao et al., 2022a). This is the main mechanism of GPX4 to prevent lipid peroxidation and inhibit ferroptosis. Inhibition of GPX4 has been shown to be a promising new cancer treatment. Down-regulation of GPX 4 inhibits tumor growth through SREBP1 signaling in oral cancer (Fukuda et al., 2021). Bupivacaine can suppress BC cell growth by decreasing GPX4 (Hao, Zhang & Huang, 2022). The degradation of the GPX4 protein induced by Fin56 can also suppress BC cell proliferation (Sun et al., 2021b). Therefore, GPX4 appears to be a promising therapy target for BC.

In this study, we investigated the potential functional role of PSMD14 in the proliferation of BC cells. Our results showed that PSMD14 overexpression promoted BC cell proliferation. Silencing of PSMD14 suppressed tumor cell growth, and was associated with down-regulation of GPX4.

## Materials and Methods

### Tissue samples and clinic data

From 2016 to 2018, 181 patients diagnosed with primary BC at the Affiliated Hospital of Qingdao University were recruited. All patients underwent surgical resection. The paraffin-embedded BC specimens and adjacent non-tumor tissues from these patients were collected. Clinicopathological data were collected as previously described (Zhang et al., 2018). Patients were followed up until July 2022, and data relative to patient prognosis were recorded. The protocol for this study and the written consent forms were approved by the Ethics Committee of the Affiliated Hospital of Qingdao University (QYFYWZLL 27227). All patients/guardians gave their written consent.

### Plasmid construction and lentivirus transduction

The lentiviral vector containing the PSMD14 sequence encoding PSMD14 overexpression (PSMD14-OE), PSMD14 knockdown (PSMD14-KD), and the control vector (PSMD14-Ctrl) were purchased from Hanheng BioChem Corporation (Shanghai, China). The plasmids were transfected into T24 and 5637 cells according to the manufacturer’s instructions. The transfection efficiency was monitored by western blotting. Cells were then infected with lentiviruses and selected by puromycin (p8230; Solarbio, Beijing, China).

### Cell lines and cultures

Human BC T24, 5637, J82, and UM-UC3 cell lines were obtained from the Cell Bank of the Chinese Academy of Sciences (Shanghai, China). Cells (except for 5637 cells) were cultured with Dulbecco’s modified Eagle’s medium (DMEM; Gibco, Waltham, MA, USA; C11995500BT; Thermo Fisher, Waltham, MA, USA) supplemented with 10–20% fetal bovine serum (FBS; Gibco, Waltham, MA, USA; 10099-141c; Thermo Fisher, Waltham, MA, USA). The 5637 cells were cultured with PRIM1640 (Gibco, Waltham, MA, USA; C11875500BT; Thermo Fisher, Waltham, MA, USA). All cells were housed in a humidified atmosphere at 37 °C with 5% CO_2_. The ferroptosis inhibitor ferrostatin-1 and ferroptosis activator RSL3 were purchased from Med ChemExpress (MCE).

### Quantitative real-time PCR analysis

Cells were lysed with TRIzol reagent (CW0580S; CWBIO, Beijing, China), and total RNA was extracted. RNA (1000 ng) was reversely transcribed and amplified using a SuperRT one-step real-time PCR kit (CW0742S; CWBIO, Beijing, China) and a real-time PCR detection system (ABI, 7500, Thermo Fisher, Waltham, MA, USA) according to the manufacturer’s protocol. GAPDH was considered the standard internal reference. Real-time PCR conditions were as follows: initial denaturation at 95 °C for 5 min and 40 cycles of 95 °C for 15 s, 65 °C for 30 s, 72 °C for 30 s, and a final extension of 1 min. The experiment was repeated three times independently. Relative quantitative method (2^−ΔΔCt^) was utilized to calculate the results, which were presented as fold changes. The designed primers were as follows: GPX4: 5′-GAGGCAAGACCGAAGTAAACTAC-3′ (F) and 5′-CCGAACTGGTTACACGGGAA-3′(R); GADPH: 5′-CTGACTTCAACAGCGACACC-3′ (F), and 5′-TGCTGTAGCCAAATTCGTTGT-3′(R).

### Western blotting analysis

Cells were lysed in radioimmunoprecipitation assay buffer (RIPA) (R0020, Solarbio, Beijing, China) supplemented with a protease inhibitor cocktail (CW2200S, CWBIO, Beijing, China) and phosphatase inhibitors (CW2383S, CWBIO, Beijing, China) for 30 min. Protein concentration was determined using a BCA protein assay kit (Solarbio, Beijing, China). Total proteins (30 μg) were separated using 12.5% SDS-PAGE and transferred to a polyvinylidene fluoride membrane (PVDF) (IPVH00010; Millipore, Burlington, MA, USA) at 110 voltage for 90 min. After blocking with 5% fat-free milk for 2 h at room temperature, the membranes were incubated at 4 °C overnight with primary antibodies against PSMD14 (1:1,000; clone: 12059-1-AP; Proteintech, Wuhan, China), GPX4 (1:1,000; clone: 67763-1-lg; Proteintech, Wuhan, China), and anti-actin rabbit polyclonal (1:5,000; clone: E-AB-2058; Elabscience, Wuhan, China).

### Cell proliferation assay

Cells were seeded at a density of 1 × 10^3^ cells per well in 96-well plates. Cell proliferation was evaluated using the CCK-8 (cell counting kit-8) assay (Dojindo, Dalian, China) at indicated time points according to the manufacturer’s instructions. Absorbance at 450 nm was measured after incubation with 10 μL CCK-8 regent and 90 μL cell culture medium for 1.5 h at 37 °C. Light absorbance was measured using an automated microplate reader (Infinit f200; Tecan, Port Melbourne, Australia).

### Colony formation assay

Cells (8 × 10^2^ cells/well) were seeded in 6-well plates. The medium was replaced every 3 days. After culturing for 14 days, the plates were washed twice with PBS, fixed with 4% paraformaldehyde for 30 min, and stained with 0.1% crystal violet for 1 min and counted.

### Immunohistochemistry

Immunohistochemistry (IHC) was performed on paraffin-embedded BC tissues and adjacent tissues (*n* = 181). After deparaffinization and rehydration by xylene and a series of graded ethanol washes, sections were treated with Tris-EDTA (pH = 9.0) at 100 °C for 10 min. The sections were then treated with 3% hydrogen peroxidase (10 min, room temperature), followed by incubation with primary antibody targeting PSMD14 (1:600; clone: 12059-1-AP; Proteintech, Wuhan, China) and GPX4 (1:800; clone: 67763-1-lg; Proteintech, Wuhan, China), for 1 h at 37 °C. Sections were incubated with HRP-conjugated secondary antibody for 60 min and a 3,3-diaminobenzidine (DAB) kit (ZL1-9017; ZSGB-Bio, Beijing, China) for 1 min. Finally, the sections were counterstained with hematoxylin.

The scoring of the IHC staining was based on the extent of positive tumor cells and the intensity of the staining (0 = negative, 1 = weak, 2 = medium, or 3 = strong). The percentage of positive tumor cells was scored as 0 (negative), 1 (1–25%), 2 (26–50%), 3 (51–75%) and 4 (>75%). The two scores were multiplied and the resulting immune-reactive score (IRS) (values from 0–12) were used to classify the samples into two categories: high (5–12 score) and low (1–4 score) (Zhang et al., 2018).

### Animal experiments

Twenty female BALB/c nude mice (4–6-weeks old, 15 g) were purchased from Beijing Vital River Laboratory Animal Technology Co., Ltd. (Beijing, China). All mice were housed under specific pathogen-free conditions in 12/12 cycle of light at room temperature (24–26 °C). Mice were fed a full fat diet and autoclaved water. The number of mice did not exceed five per cage. A total of 1 × 10^7^ infected 5637 cells were suspended in 100 μL PBS and injected into the shoulder of the mice. Tumor length (L) and width (W) were observed for 4 weeks. Tumor volume (V) was monitored by measuring the length and width of the tumor using the following equation: V = (L × W^2^) × 0.5. The mice were euthanized by cervical dislocation after inhalational of CO_2_ when the maximum diameter of any tumor was near 1.5 cm. Tumor tissues were excised and embedded in paraffin for ematoxylin and eosin (HE) or IHC staining. Animal experiments were reviewed and approved by the Ethics Committee on Animal Experiments of the affiliated hospital of Qingdao University (AHQU-MAL20220715).

### Statistical analysis

The results were analyzed with SPSS 19.0.0 (SPSS, Chicago, IL, USA). Chi-square (χ2) or Fisher’s exact test were used to compare frequencies between groups. Survival curves for disease-free survival (DFS) and overall survival (OS) were calculated using Kaplan-Meier analysis with the log-rank test. Multivariate analysis was performed using the Cox proportional hazard model. A *P*-value < 0.05 was considered statistically significant.

## Results

### PSMD14 status and association with clinicopathological characteristics

**Figure 1:**
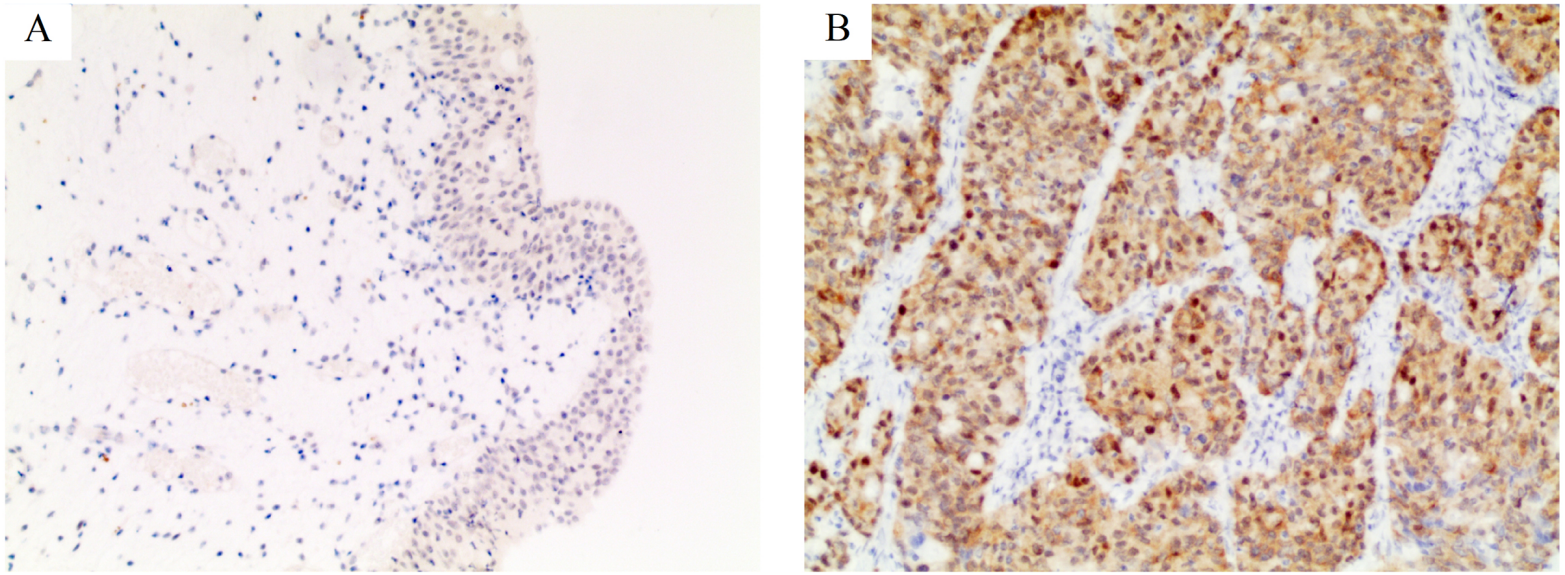
Immunohistochemical staining for PSMD14 in bladder cancer. The intensity of staining was scored as negative (0, A) in normal bladder tissues and strong (3+, B) in bladder cancer. All images are at 100× magnification.

We evaluated the expression of PSMD14 in 181 pairs of BC and corresponding normal tissues by IHC. IHC staining showed that the PSMD14 protein was expressed at significantly higher levels in BC (138/181) compared to normal tissues (43/181) (76.24% *vs* 23.76%, *P* = 0.02) (Fig. 1). The correlation between PSMD14 expression and clinicopathological characteristics is presented in Table 1. PSMD14 showed significantly higher expression in tumors with larger diameters (tumor diameter >3 cm *vs* tumor diameter ≤3 cm; 85.14% *vs* 70.09%, *P* = 0.019). Patients with a family history of cancer were more likely to have high PSMD14 expression (yes *vs* no; 92.16% *vs* 70.00%, *P* = 0.002). Although high expression of PSMD14 was more frequent in patients with tumor vascular invasion, there was no statistical significance (92.86% *vs* 74.85%, *P* > 0.05). No significant differences were found between PSMD14 expression and other clinicopathological characteristics in this study (Table 1).

**Table 1:** Correlations between PSMD14 expression and clinicopathological characteristics (*n* = 181).

		PSMD14 expression		
Characteristics	Number	High expression (%)	Low expression (%)	*P*
Gender				
Male	129	101 (78.29)	28 (21.71)	0.307
Female	52	37 (71.15)	15 (28.85)	
Age (year)				
≤60	66	47 (71.21)	19 (28.79)	0.228
>60	115	91 (79.13)	24 (20.87)	
Tumor invasion (T)				
Yes	103	78 (75.73)	25 (24.27)	0.852
No	78	60 (76.92)	18 (23.08)	
Tumor stage				
I	72	54 (75.00)	18 (25.00)	0.899
II	80	61 (76.25)	19 (23.75)	
III	29	23 (79.31)	6 (20.69)	
Tumor diameter				
≤3 cm	107	75 (70.09)	32 (29.91)	0.019
>3 cm	74	63 (85.14)	11 (14.86)	
Bowel wall invasion (T)				
T1+T2	97	72 (74.23)	25 (25.77)	0.493
T3+T4	84	66 (78.57)	18 (21.43)	
Lymph node metastasis (N)				
With	23	19 (82.16)	4 (17.39)	0.443
Without	158	119 (75.32)	39 (24.68)	
Lymphovascular invasion				
Yes	21	17 (80.95)	4 (19.05)	0.786*
No	160	121(75.63)	39 (24.38)	
Vascular invasion				
Yes	14	13 (92.86)	1 (7.14)	0.193*
No	167	125 (74.85)	42 (25.15)	
Alcohol intake history				
Ever	78	60 (76.92)	18 (23.08)	0.852
Never	103	78 (75.73)	25 (24.27)	
Smoking history				
Ever	40	29 (72.50)	11 (27.50)	0.529
Never	141	109 (77.30)	32 (22.70)	
Cancer family history				
Yes	51	47 (92.16)	4 (7.84)	0.002
No	130	91 (70.00)	39 (30.00)	

**Note:**

Fisher’s exact test was used.

### Prognostic value of PSMD14 expression in primary BC

Kaplan-Meier survival analysis and log rank test showed that factors significantly associated with DFS were sex (*P* = 0.002), invasive cancer (*P* = 0.001), tumor stage (*P* = 0.001), invasion of the intestinal wall (*P* = 0.001), lymph node metastasis (*P* = 0.001), vessel carcinoma embolus (*P* = 0.001), vascular invasion (*P* = 0.001), alcohol intake (*P* = 0.001), smoking history (*P* = 0.0012), family history of cancer (*P* = 0.0436), and PSMD14 expression (*P* = 0.0232) (Table 2).

**Table 2:** Univariate analysis of prognostic factors influencing disease free survival (DFS) in stage I–III bladder cancer.

Disease-free survival				
Characteristics		HR	95% CL	*P*
Gender	Male *vs* female	3.155	[1.739–5.726]	0.002
Age (year)	≤60 *vs* >60	1.381	[0.814–2.342]	0.231
Tumor invasion	Yes *vs* no	3.147	[1.882–5.262]	0.001
Tumor stage	I *vs* II *vs* III			0.001
Tumor diameter	≤3 cm *vs* >3 cm	1.17	[0.695–1.973]	0.552
Bowel wall invasion (T)	T3+T4 *vs* T1+T2	4.967	[2.930–8.422]	0.001
Lymph node metastasis (N)	Yes *vs* no	11.48	[4.68–28.16]	0.001
Lymphovascular invasion	Yes *vs* no	12.9	[5.052–32.96]	0.001
Vascular invasion	Yes *vs* no	15.74	[4.952–50.03]	0.001
Alcohol intake	Yes *vs* no	3.192	[1.878-5.425]	0.001
Smoking	Yes *vs* no	2.939	[1.530–5.645]	0.0012
Cancer family history	Yes *vs* no	1.828	[1.018–3.285]	0.0436
PSMD14 expression	High *vs* low	1.961	[1.096–3.506]	0.0232

**Figure 2:**
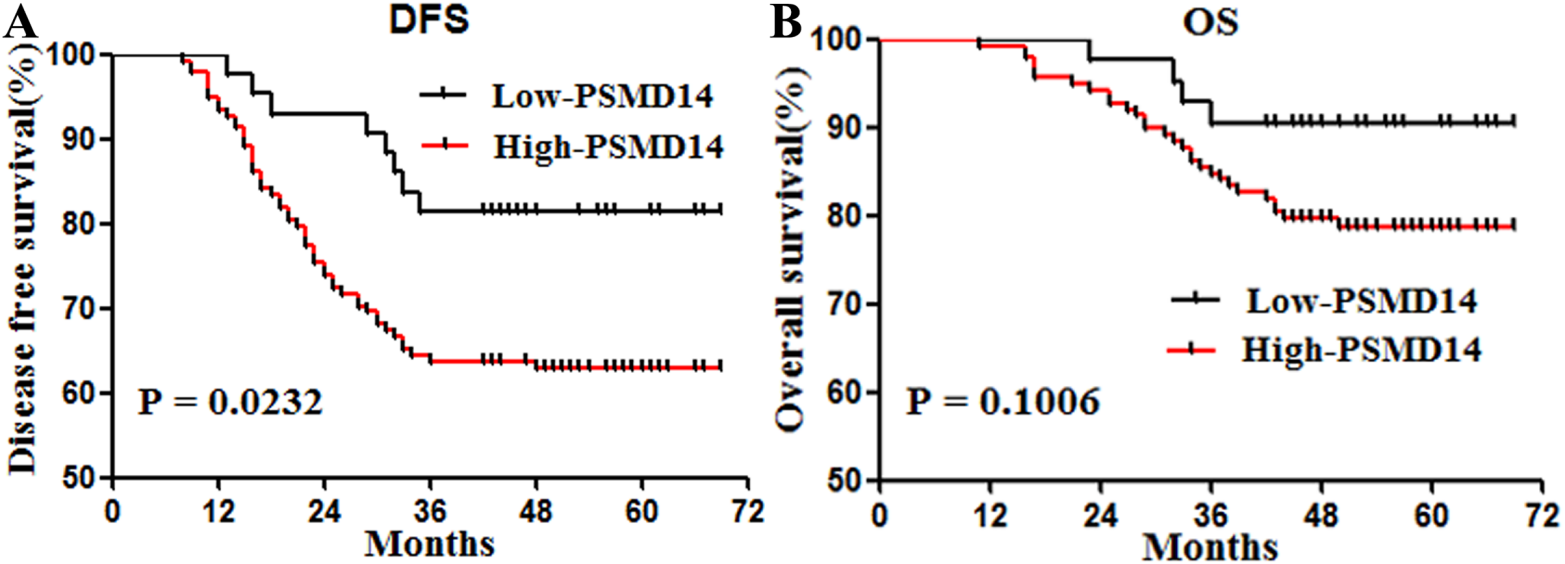
Survival curves for disease free survival (DFS) and overall survival (OS) in stage I–III bladder cancer according to PSMD14 expression. (A) DFS according to PSMD14 expression: PSMD14 expression was a predicted factor for worse DFS. (B) OS according to PSMD14 expression: PSMD14 was not a statistically significant risk factor for OS.

Patients with high expression of PSMD14 showed shorter DFS (*P* = 0.0232) (Fig. 2A). To determine prognostic values independent of sex, we entered invasive cancer, tumor stage, intestinal wall invasion, lymph node metastasis, vessel carcinoma embolus, vascular invasion, history of alcohol intake, smoking history, cancer family history and expression information of PSMD14 in a Cox regression model. We found that PSMD14 expression was an independent predictor of worse DFS, and tumors with high expression of PSMD14 were associated with a 2.89-fold increase in the risk of cancer recurrence or metastasis (HR = 2.89, 95% CI [1.247–6.711], *P* = 0.013) (Table 3). In addition, PSMD14 was not a statistically significant risk factor for OS in the univariate analysis (Table 3).

**Table 3:** Independent prognostic factors correlating with disease free survival (DFS) in stage I–III bladder cancer using Cox proportional hazard model analysis.

Characteristics		HR	95% CL	*P*
Gender	Male *vs* female	1.828	[0.900–3.713]	0.095
Tumor invasion	Yes *vs* no	1.321	[0.382–4.566]	0.66
Tumor stage	I *vs* II *vs* III	2.221	[0.994–4.961]	0.149
Bowel wall invasion (T)	T3+T4 *vs* T1+T2	0.394	[0.138–1.124]	0.082
Lymph node metastasis (N)	Yes *vs* no	1.189	[0.373–3.787]	0.769
Lymphovascular invasion	Yes *vs* no	0.575	[0.269–1.226]	0.152
Vascular invasion	Yes *vs* no	1.625	[0.605–4.365]	0.335
Alcohol intake history	Yes *vs* no	2.16	[1.212–3.846]	0.009
Smoking history	Yes *vs* no	0.662	[0.356–1.233]	0.194
Cancer family history	Yes *vs* no	1.337	[0.676–2.654]	0.404
PSMD14 expression	High *vs* low	2.89	[1.247–6.711]	0.013

### PSMD14 promoted BC cell proliferation *in vitro* and *in vivo*

**Figure 3:**
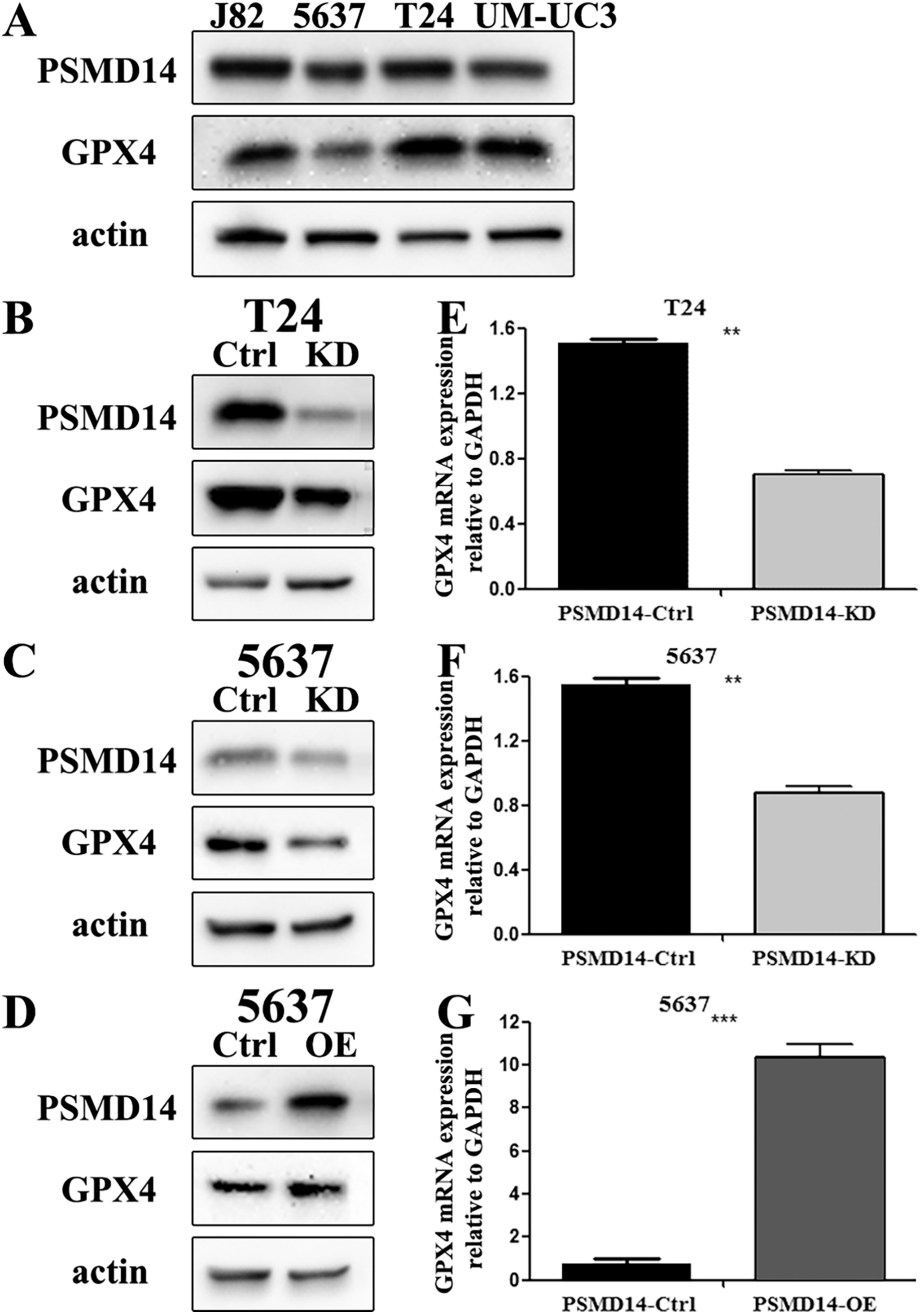
The knockdown and over-expression efficiency of PSMD14 in bladder cancer cell lines. (A) Western blot analysis of PSMD14 and GPX4 protein expression in T24, 5637, J82, and UM-UC3 cell lines. (B and E) The knockdown efficiency of PSMD14-shRNA in protein (B) and mRNA (E) level of T24 cells. (C and F) The knockdown efficiency of PSMD14-shRNA in protein (C) and mRNA (F) level of 5637 cells. (D) PSMD14 protein was significantly up-regulated in 5637 cells by PSMD14 overexpression plasmid. (G) PSMD14 mRNA was significantly up-regulated in 5637 cells by PSMD14 overexpression plasmid. Actin and GAPDH were used as the control group. All experiments were repeated at least three times. **P < 0.01; ***P < 0.001.

**Figure 4:**
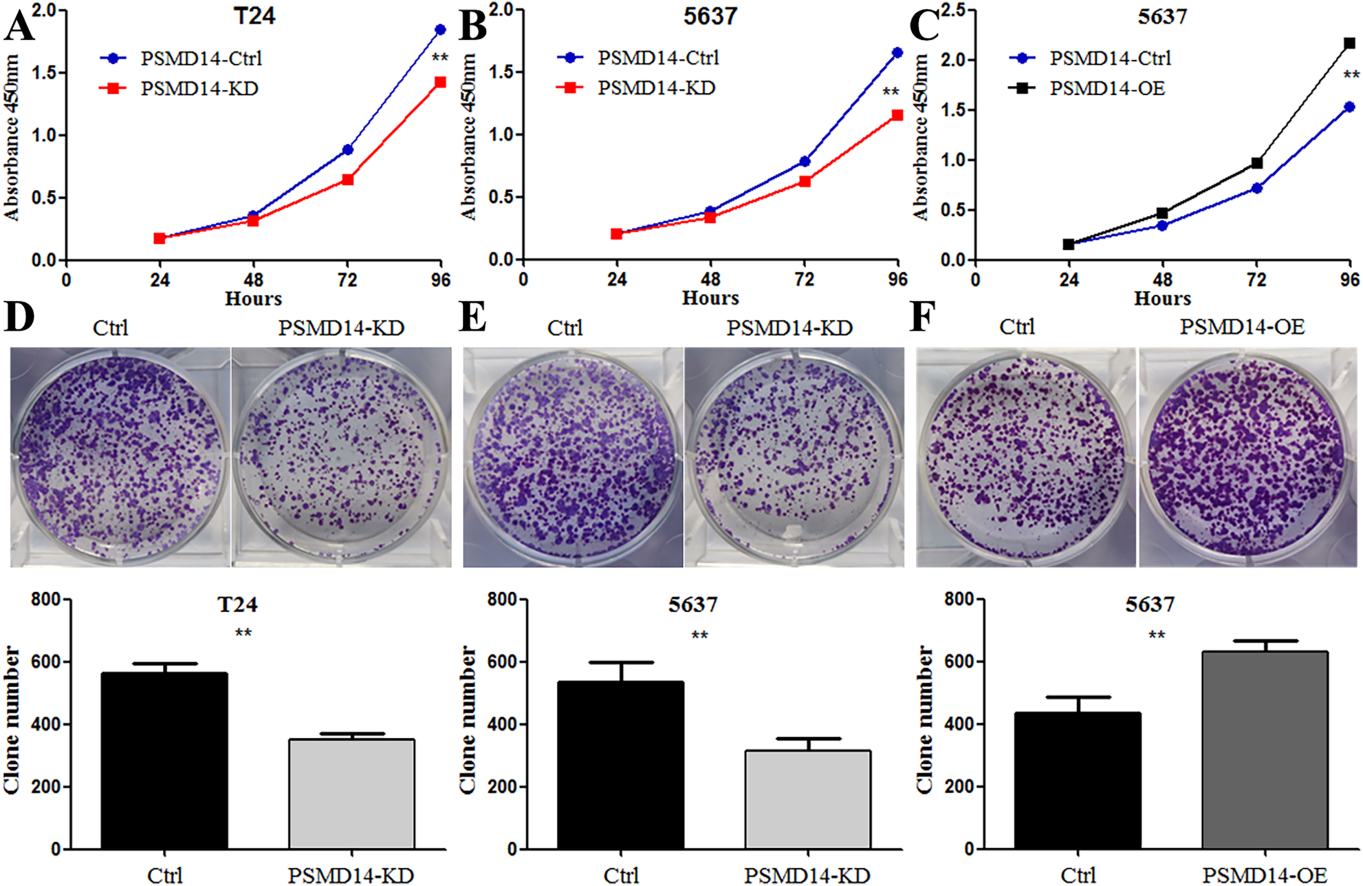
Knockdown of PSMD14 inhibits bladder cancer cells proliferation *in vitro*. Knockdown of PSMD14 decreased cell viability compared to control group in a time-dependent manner at 24, 48, 72, and 96 h measured at 450 nm absorbance by CCK8 assay in T24 and 5637 cell lines (A and B). (C) PSMD14 over-expression increased cell viability compared to control group in a time-dependent manner. (D–F) The colony formation assay showed that PSMD14 knockdown decreased the number of colonies and PSMD14 over-expression increased the number of colonies. All experiments were repeated at least three times. **P < 0.01.

To assess the functional role of PSMD14 in the development and progression of BC, we first examined endogenous levels of PSMD14 in several BC cell lines. We found high endogenous expression of PSMD14 in T24, UM-UC3 and J82, and low expression levels in 5637 cells (Fig. 3A). We performed a knockdown of PSMD14 expression in T24 (Figs. 3B and 3E) and 5637 cells (Figs. 3C and 3F) using the PSMD14-KD vector. BC cell proliferation with low expression of PSMD14 was significantly suppressed compared to control cells (Figs. 4A and 4B). Similarly, the colony formation assay showed that PSMD14 depletion inhibited colony number formation (Figs. 4D and 4E). Conversely, PSMD14 was overexpressed in 5637 cells (Figs. 3D and 3G). Compared to PSMD14-Ctrl cells, the overexpression of PSMD14 promoted BC cell growth according to the CCK8 assay (Fig. 4C). The colony formation assay showed that PSMD14 overexpression increased the colony number of 5637 cells (Fig. 4F). Therefore, the reciprocal effects of knockdown and overexpression of PSMD14 *in vitro* suggested that PSMD14 promoted the proliferation of BC cells.

**Figure 5:**
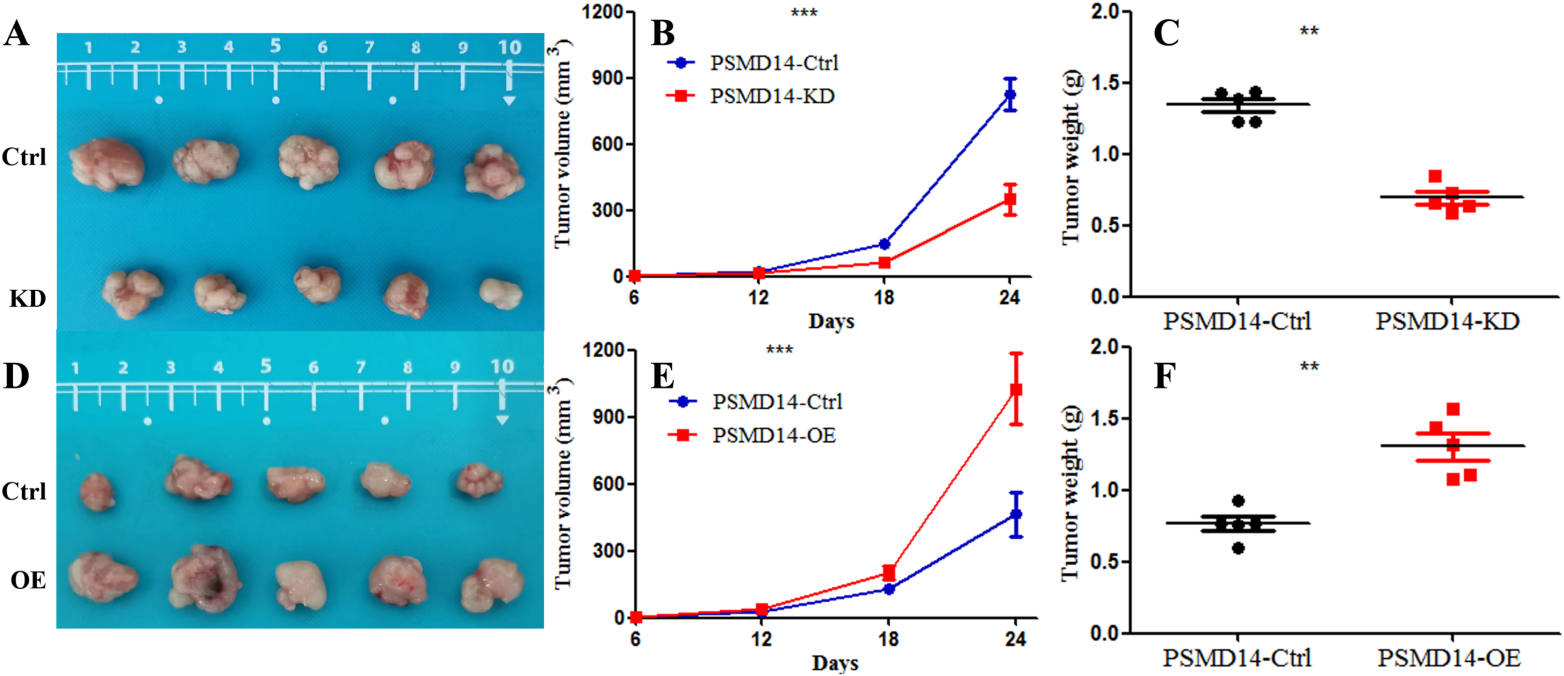
Knockdown of PSMD14 inhibits tumor growth in nude mice. Briefly, 1 × 10^7^ 5637 ^PSMD14-KD^ cells, 5637 ^PSMD14-Ctrl^ cells or 5637 ^PSMD14-OE^ cells suspended in 100 μL phosphate-buffered saline were injected into the shoulder of the nude mice (*n* = 5 per group). As shown in (A–C), tumors formed by PSMD14-KD cells were significantly smaller than those formed by the control cells (824.4 ± 160.67 mm^3^ *vs* 349.2 ± 151.14 mm^3^, *P* = 0.001). Tumor volume (463.4 ± 222.27 mm^3^ *vs* 1,026.4 ± 351.65 mm^3^, *P* = 0.002) and weight (0.766 ± 0.12 g *vs* 1.304 ± 0.21 g, *P* = 0.002) were notably increased in the PSMD14-OE group compared with the control group (D–F). **P < 0.01; ***P < 0.001.

**Figure 6:**
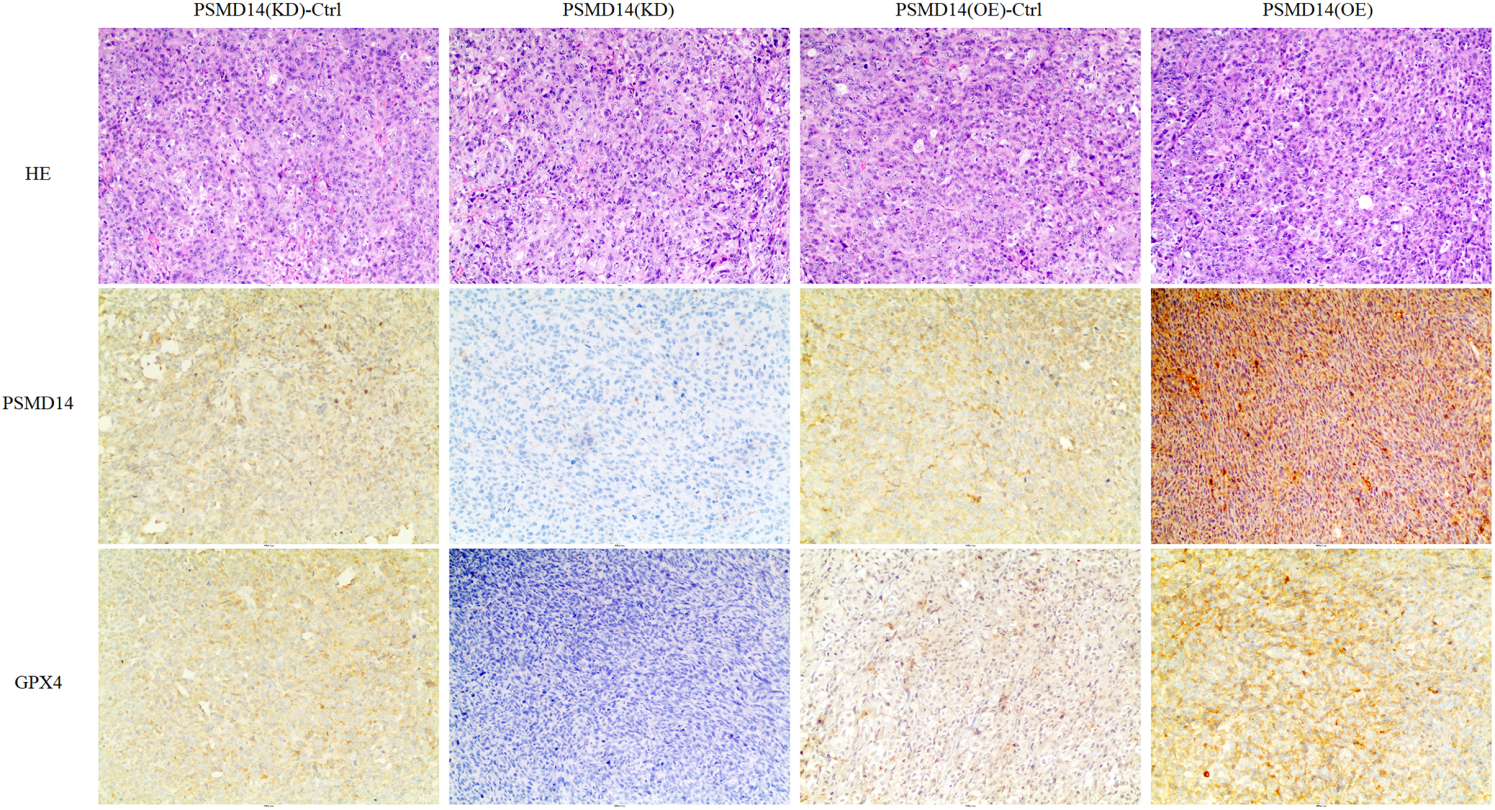
IHC analysis for PSMD14 and GPX4 expression in PSMD14-KD and PSMD14-OE tumor tissues. The nude mice were euthanized when the maximum diameter of any tumor was near 1.5 cm. Tumor tissues were excised and embedded in paraffin for hematoxylin and eosin staining and immunohistochemical (IHC) staining. IHC analysis demonstrated that PSMD14 expression increased in the PSMD14-OE group and decreased in the PSMD14-KD group compared with the control group. GPX4 expression was up-regulated in PSMD14-OE tumor tissue, and GPX4 expression was down-regulated in PSMD14-KD tumor tissue. All images are at 100× magnification.

A total of 20 mice were used to evaluate the proliferation potential of PSMD14 *in vivo*. Briefly, 5637 cells transfected with different vectors (PSMD14-OE and PSMD14-KD) were injected subcutaneously into the nude mice. Cells transfected with the PSMD14-Ctrl vector were injected as a control. The tumors were formed on day 6 after injection and were collected on day 24. As shown in Fig. 5, the tumors formed by PSMD14-KD cells were significantly smaller than those formed by the control cells (824.4 ± 160.67 mm^3^ *vs* 349.2 ± 151.14 mm^3^, *P* = 0.001). Tumor volume (463.4 ± 222.27 mm^3^ *vs* 1,026.4 ± 351.65 mm^3^, *P* = 0.002) and weight (0.766 ± 0.12 g *vs* 1.304 ± 0.21 g, *P* = 0.002) were markedly increased in the PSMD14-OE group compared to the control group. The tumors were collected and further analyzed using IHC. IHC analysis demonstrated that PSMD14 expression increased in the PSMD14-OE group and decreased in the PSMD14-KD group compared to the control group (Fig. 6).

### Depletion of PSMD14 antagonizes BC growth by decreasing the expression of the GPX4 protein

**Figure 7:**
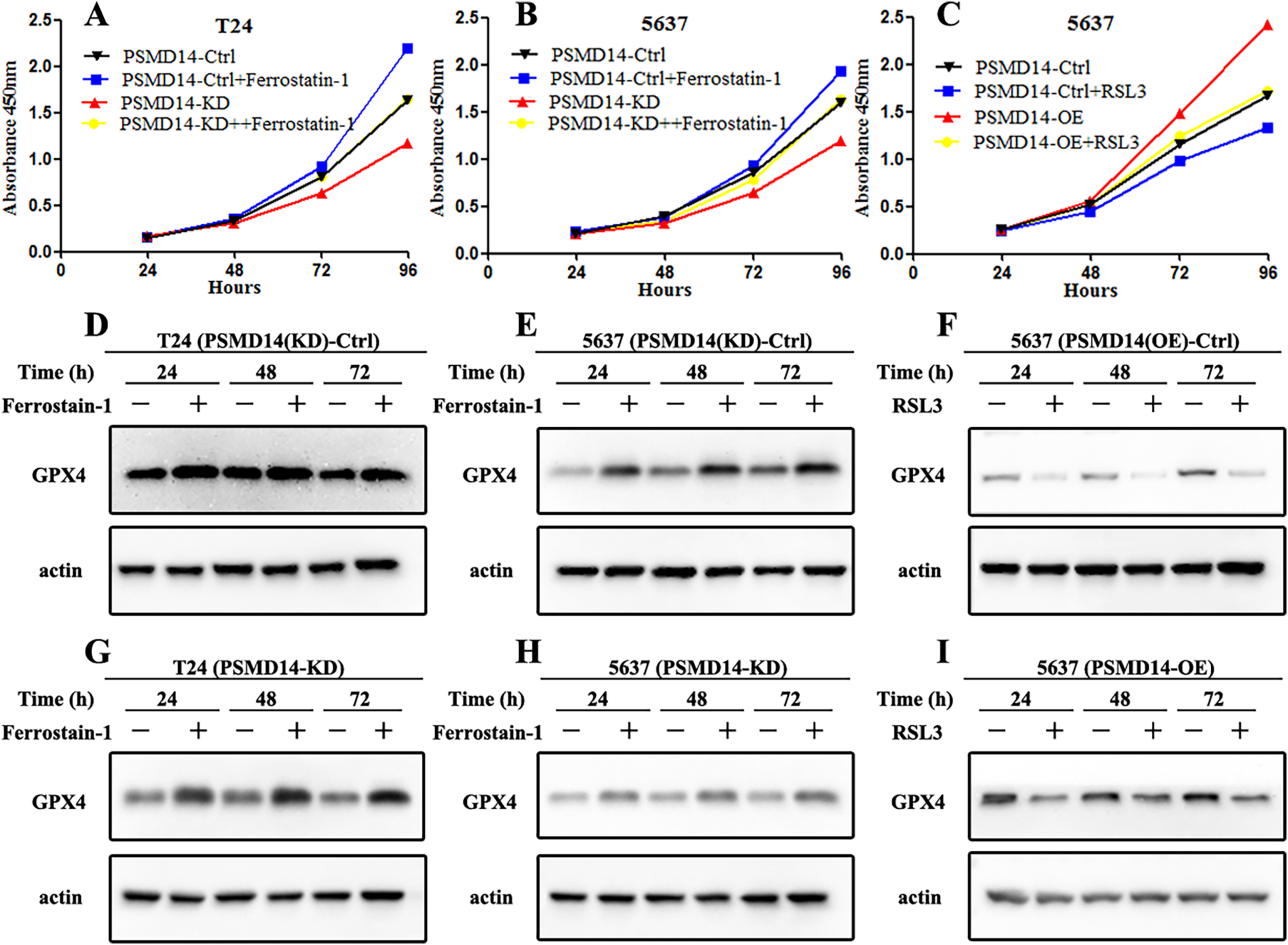
Depletion of PSMD14 suppresses bladder cancer proliferation by decreasing GPX4. (A and B) The inhibition of cell proliferation induced by PSMD14 depletion was reversed when cells were treated with ferrostatin-1, as determined by the CCK-8 assay. (C) The promotion of cell proliferation induced by PSMD14 overexpression was reversed when cells were treated with RSL3, as determined by the CCK-8 assay. (D and E) Western blot analysis showing the expression levels of GPX4 in PSMD14 (KD)-control cells treated with ferrostatin-1 in a time-dependent manner at 24, 48, and 72 h. (F) Western blot analysis showing the expression levels of GPX4 in PSMD14 (OE)-control cells treated with RSL3 in a time-dependent manner at 24, 48, and 72 h. (G and H) Western blot analysis showing the expression levels of GPX4 in PSMD14-KD cells treated with ferrostatin-1 in a time-dependent manner at 24, 48, and 72 h. (I) Western blot analysis showing the expression levels of GPX4 in PSMD14-OE cells treated with RSL3 in a time-dependent manner at 24, 48, and 72 h. All experiments were repeated at least three times.

GPX4, a ferroptosis-related gene in mammals, plays a pivotal role in the inhibition of various tumors. To determine whether PSMD14 acts on GPX4 in BC, PCR, western blotting, and IHC assays were performed to evaluate the status of GPX4 in PSMD14-OE and PSMD14-KD BC cells. We found that GPX4 was markedly down-regulated in PSMD14-KD BC cells, and GPX4 was markedly up-regulated in PSMD14-OE BC cells (Figs. 3B–3D). To verify whether PSMD14 regulated BC proliferation through the GPX4 pathway, we treated PSMD14-OE BC cells with RSL3 and PSMD14-KD cells with ferrostatin-1. As expected, the promotion of cell proliferation induced by PSMD14 overexpression was reversed when BC cells were treated with RSL3, as shown by the CCK-8 assay (Fig. 7C). The inhibition of PSMD14-KD-induced cell proliferation was also reversed when treated with ferrostatin-1 (Figs. 7A and 7B). Western blotting assays showed that GPX4 expression was significantly increased in ferrostatin-1 treated cells, and GPX4 was significantly decreased in RSL3 treated cells (Fig. 7). Together, these results indicated that the depletion of PSMD14 inhibited BC tumor growth *in vitro* and *in vivo* by targeting GPX4.

## Discussion

Ubiquitination and deubiquitination play vital roles in cell growth, proliferation, and survival (Han et al., 2022). Ubiquitination regulation is important not only for transcription and post-translation, but also for protein levels. Specifically, the highly coordinated interaction between ubiquitination/deubiquitination maintains the proper protein levels required for normal cell function (Ciechanover, 2015; Husnjak & Dikic, 2012). Coordination of ubiquitination and deubiquitination maintains cell stability through the degradation of unnecessary or harmful components. When ubiquitination/deubiquitination regulation is blocked, the defunct biological processes may subsequently induce serious human diseases, such as tumors (Deng et al., 2020). The ubiquitination-proteasome system has attracted increasing attention in the treatment of cancer in recent years (Gavali et al., 2021; Hu et al., 2021). There is increasing evidence that points to the importance of PSMD14 in tumor development and progression. However, data on PSMD14 in patients with BC have been limited. Therefore, we conducted this study to evaluate the function of PSMD14 in BC. Furthermore, we analyzed possible associations between PSMD14 expression, clinicopathological characteristics, and prognostic role in patients with primary BC. Our results showed that PSMD14 depletion can inhibit BC tumor cell growth by decreasing GPX4, and high expression of PSMD14 is an independent risk factor for worse DFS in BC.

Previous studies have demonstrated that PSMD14 is involved in the ubiquitin proteasome system that degrades various intracellular proteins (Seo et al., 2019; Zhang, Zhang & Chen, 2022). The high expression of PSMD14 has been shown to exert protoneoplastic effects in many cancer cells, including liver cancer, CRC, and esophageal squamous cell carcinoma (Lv et al., 2020; Wang et al., 2018; Zhu et al., 2018). In our study, IHC findings revealed that PSMD14 was upregulated in patients with BC. In addition, we analyzed the association between the status of PSMD14 and various patient- and tumor-specific parameters to evaluate which patients might present with high expression of PSMD14. The most significant finding was that the expression of PSMD14 was significantly correlated with increasing tumor diameter. We also found that the expression of PSMD14 was an independent risk factor for worse DFS in patients with BC. Therefore, these clinical data suggested that tumors with PSMD14 overexpression in BC patients were significantly correlated with aggressive tumor characteristics. Multiple studies have confirmed that there are noticeable family genetic characteristics in the tumorigenesis of malignant tumors, such as ovarian cancers (BRCA1 and BRCA2 mutations) (Heemskerk-Gerritsen et al., 2022), Li-Fraumeni syndrome (TP53 mutation) (Rocca et al., 2022) and hereditary nonpolyposis colorectal cancer (DNA mismatch repair deficient) (Liotta et al., 2021). Similarly, our study showed that patients with a family history of cancer were more likely to have high PSMD14 expression. This result illustrates that up-regulation of PSMD14 has a notable family aggregation characteristic and genetic tendency.

To further investigate the role of PSMD14 in BC, we engineered a stable knockdown of PSMD14 expression by lentivirus-mediated siRNA in human BC cell lines T24 and 5637. Knockdown of PSMD14 inhibits cell proliferation and colony formation. Conversely, PSMD14 overexpression promoted BC cell growth *in vitro* and *in vivo*. This result corresponded to previous studies using different cancer cell lines. Knockdown of PSMD14 upregulated the protein levels of p53 and its downstream targets such as p21, caspase-3, and Bax to mediate cell apoptosis (Wang et al., 2018). PSMD14 also promotes the progression of ovarian cancer by decreasing the enzymatic activity of PKM2 (Sun et al., 2021a). Another study conducted by Wang et al. (2019) demonstrated that down-regulation of PSMD14 decreased caveolin-1-mediated lysosomal degradation of TGFBR1 and TGFBR2 by deubiquitinating the TGF-β receptor. Lv et al. (2020) identified PSMD14 as a novel post-translational regulator of GRB2. PSMD14 could inhibit GRB2 degradation by deubiquitinating this oncoprotein in liver cancer cells (Lv et al., 2020). Therefore, all of these studies confirmed that PSMD14 acted as an oncogene in cancer.

Ferroptosis is a recently discovered type of cell death that differs from apoptosis, necrosis, and pyroptosis (Song et al., 2022). GPX4 is an important enzyme that inhibits the occurrence of ferroptosis by reducing lipid peroxide levels. Several studies have shown that GPX4 participates in carcinogenesis and plays a critical role in tumor growth, metastasis, and drug resistance (Hassannia, Vandenabeele & Berghe, 2019). Inhibition of GPX4 interferes with intracellular iron homeostasis and increases lipid peroxide levels, inducing ferroptosis and exerting anticancer effects in ovarian cancer (Li, Zhang & Chao, 2021). Sha et al. (2021) analyzed 199 breast cancer patients treated with paclitaxel-cisplatin-based neoadjuvant chemotherapy. The result showed that patients with low expression of GPX4 had a better prognosis for DFS (Sha et al., 2021). In lung cancer, GPX4 was upregulated in lapatinib resistant samples, and inhibition of GPX4 overcame resistance to lapatinib by promoting ferroptosis (Ni et al., 2021). Furthermore, Wei et al. (2020) showed that targeting GPX4 rather than GSH directly may be a more effective method to induce ferroptosis in cancer cells. Thus, inducing GPX4 inactivation is an effective anticancer strategy to inhibit tumor cell proliferation (Hassannia, Vandenabeele & Berghe, 2019), especially in BC (Hao, Zhang & Huang, 2022; Sun et al., 2021b).

Autophagy-mediated lysosomal degradation and the ubiquitin proteasome system are two major pathways that maintain intracellular homeostasis by removing damaged organelles and misfolded proteins (Xu et al., 2020). Selective autophagy also regulates ferroptosis by degrading GPX4 (Wu et al., 2022). Therefore, the co-targeting of autophagy and GPX4 could function synergistically to kill BC cells (Sun et al., 2021b). Deubiquitinating enzymes are known to be involved in various signaling pathways (*i.e*., the Wnt and TGF-β pathways) by regulating the ubiquitination status of proteins (Clague, Coulson & Urbé, 2012; Han, Lee & Han, 2017). PSMD14 could enhance cancer cells malignancy through the LRPPRC/Beclin1-Bcl-2/SQSTM1 signaling pathway inducing autophagy (Zhao et al., 2022b). Given that the crosstalk between different signal pathways may induce multiple and complicated effects, it is speculated that PSMD14 might interact with GPX4. In our study, we revealed for the first time that the knockdown of PSMD14 could inhibit the proliferation and colony formation of BC cells by targeting GPX4. This result might provide a molecular basis for new strategies targeting deubiquitination and ferroptosis-induced cell death pathways. Therefore, the deubiquitination and ferroptosis system may become an effectively targeted anticancer pathway. To further confirm the association between PSMD14 and GPX4 activity, we treated PSMD14-OE cells with RSL3 to suppress GPX4. We found that the proliferation of PSMD14-OE cells was significantly inhibited compared to control cells, and GPX4 was negatively regulated at the same time. Our data further confirmed that GPX4 was a downstream molecule of PSMD14 in BC cells. We preliminarily found that the depletion of PSMD14 inhibited the proliferation of BC cells by reducing GPX4, but the mechanism by which PSMD14 regulates GPX4 requires further study.

There were some limitations in this study. First, we only evaluated patients with early-stage BC because we used surgical specimens. Thus, the status of PSMD14 expression in advanced or recurrent BC remains unknown. Second, we only used GPX4 inhibitors and activators for the functional recovery test. Specific knockdown and overexpression of GPX4 should be conducted. Third, although previous studies have shown that PSMD14 could mediate cell apoptosis (Wang et al., 2018), we only evaluated the impact of PSMD14 on cell proliferation. Additional mechanisms and signaling pathways, including the cell cycle, lipid metabolism, deubiquitination, and EMT, should be evaluated in the future.

## Conclusions

This study demonstrated that PSMD14 is highly expressed in BC patients and that PSMD14 expression correlates with poor DFS. Depletion of PSMD14 could inhibit BC proliferation through down-regulation of GPX4. Therefore, PSMD14 may be considered an effective target for the treatment of BC.

## Supplemental Information

10.7717/peerj.14654/supp-1 Supplemental Information 1 Author checklist.

10.7717/peerj.14654/supp-2 Supplemental Information 2 Raw data for the study.

10.7717/peerj.14654/supp-3 Supplemental Information 3 Original WB pictures for Figure 3.

10.7717/peerj.14654/supp-4 Supplemental Information 4 Original WB pictures for Figure 7.

## Additional Information and Declarations

### Data Availability

The following information was supplied regarding data availability: The raw data are available in the Supplemental File.
