# Supplementary figures and images for: Depletion of PSMD14 suppresses bladder cancer proliferation by regulating GPX4
Source: PeerJ. 2023 Jan 6;11:e14654. doi: 10.7717/peerj.14654 (PMC9828270; doi:10.7717/peerj.14654)

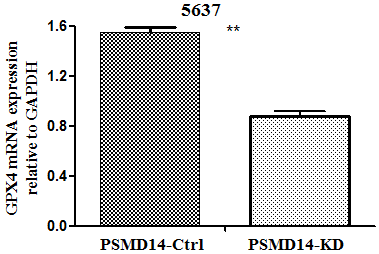

Supplement: Supplemental Information 3 [file peerj-11-14654-s003.zip › 5637-F.png]

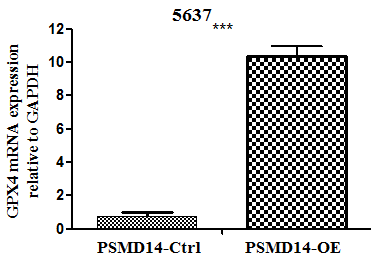

Supplement: Supplemental Information 3 [file peerj-11-14654-s003.zip › 5637-G.png]

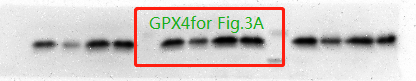

Supplement: Supplemental Information 3 [file peerj-11-14654-s003.zip › GPX4-Figure -3A-illustration.tif]

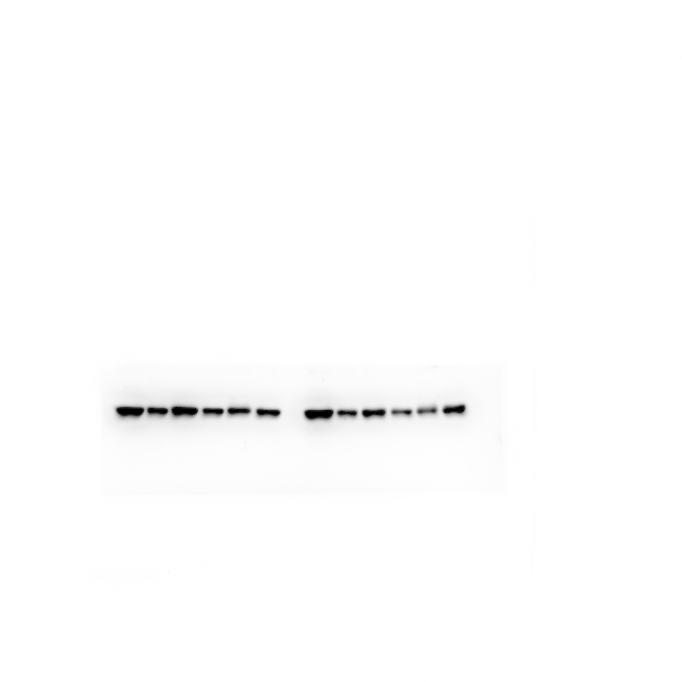

Supplement: Supplemental Information 3 [file peerj-11-14654-s003.zip › GPX4-Figure -3B.tif]

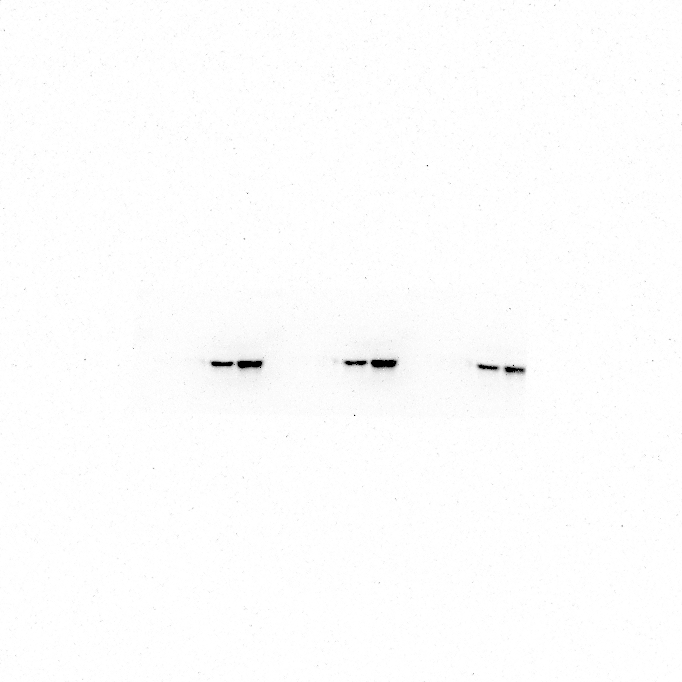

Supplement: Supplemental Information 3 [file peerj-11-14654-s003.zip › GPX4-Figure -3D.tif]

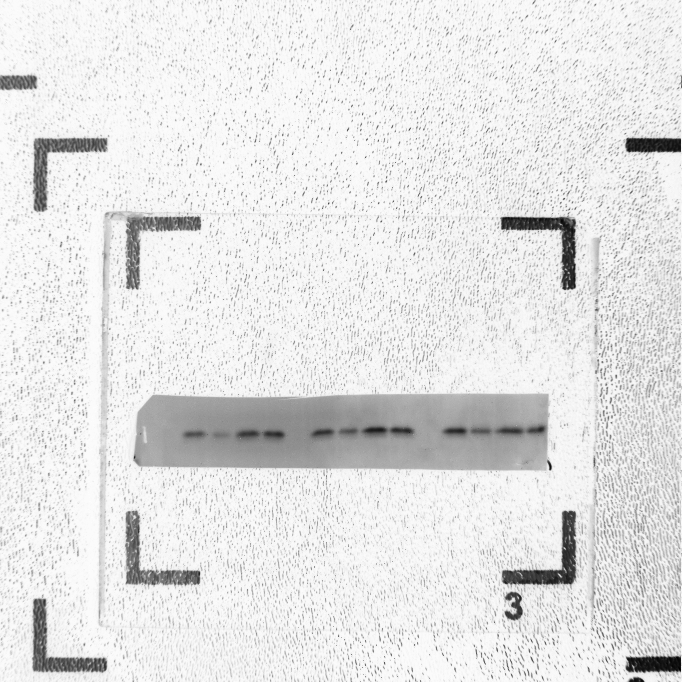

Supplement: Supplemental Information 3 [file peerj-11-14654-s003.zip › GPX4-Figure 3A-MARKER.tif]

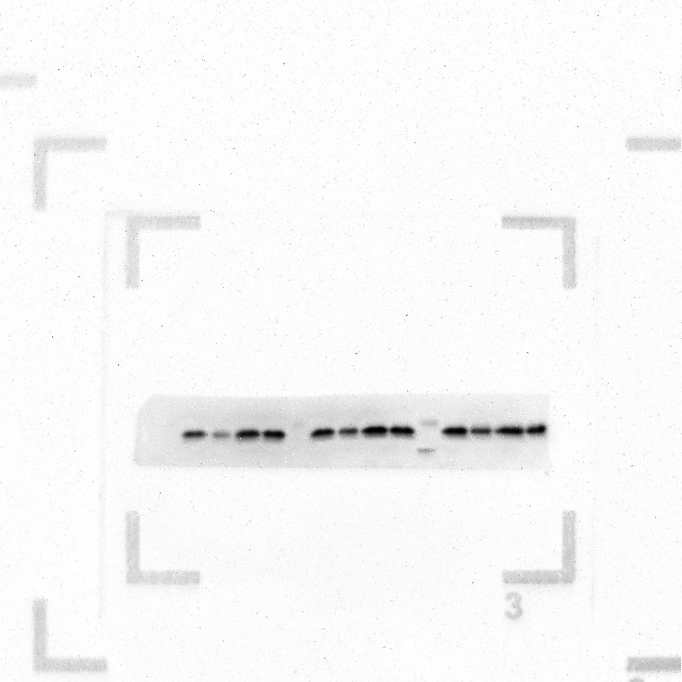

Supplement: Supplemental Information 3 [file peerj-11-14654-s003.zip › GPX4-Figure 3A.tif]

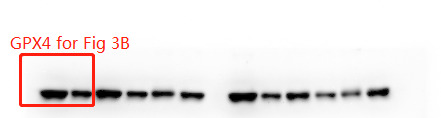

Supplement: Supplemental Information 3 [file peerj-11-14654-s003.zip › GPX4-Figure 3B-illustration.tif]

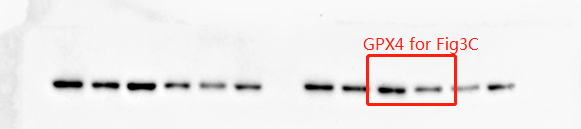

Supplement: Supplemental Information 3 [file peerj-11-14654-s003.zip › GPX4-Figure 3C-illustration.tif]

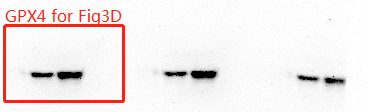

Supplement: Supplemental Information 3 [file peerj-11-14654-s003.zip › GPX4-Figure 3D-illustration.tif]

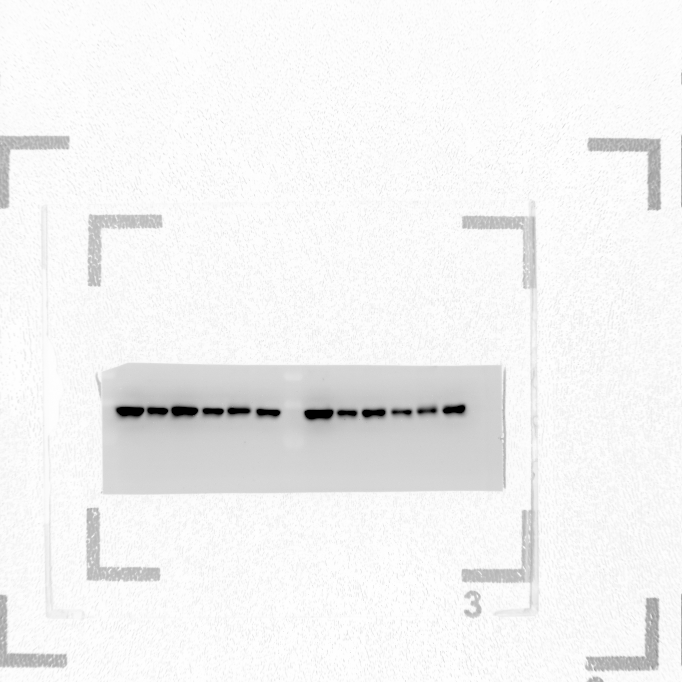

Supplement: Supplemental Information 3 [file peerj-11-14654-s003.zip › GPX4-marker-Figure -3B.tif]

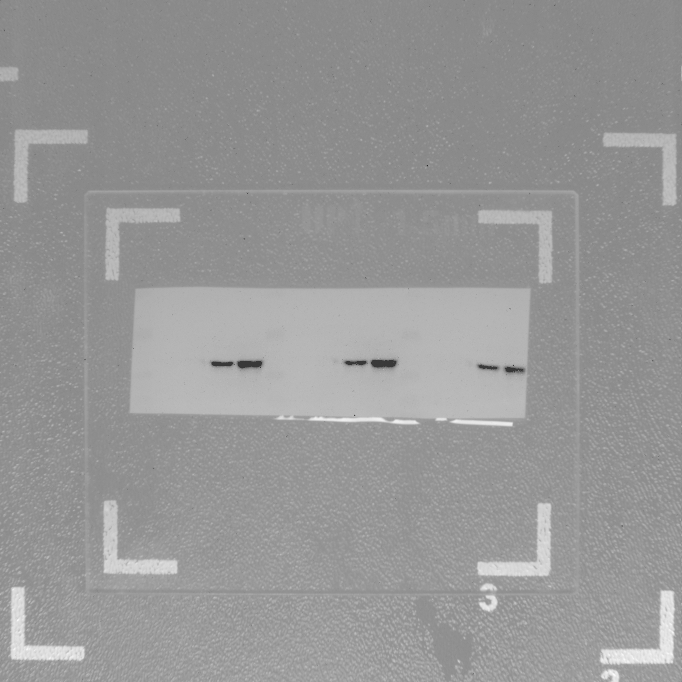

Supplement: Supplemental Information 3 [file peerj-11-14654-s003.zip › GPX4-marker-Figure -3D.tif]

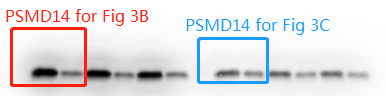

Supplement: Supplemental Information 3 [file peerj-11-14654-s003.zip › PSMD14-KD-Figure 3B-3C-illustration.tif]

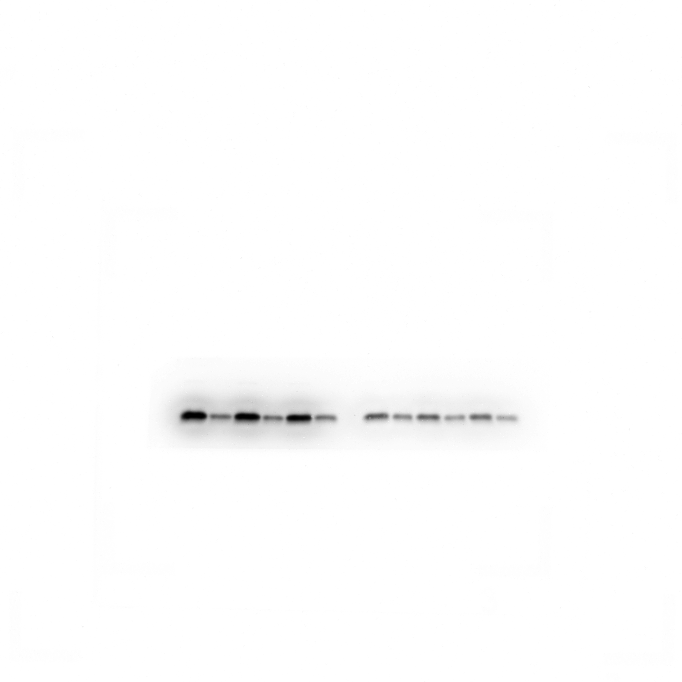

Supplement: Supplemental Information 3 [file peerj-11-14654-s003.zip › PSMD14-KD-Figure 3B-3C.tif]

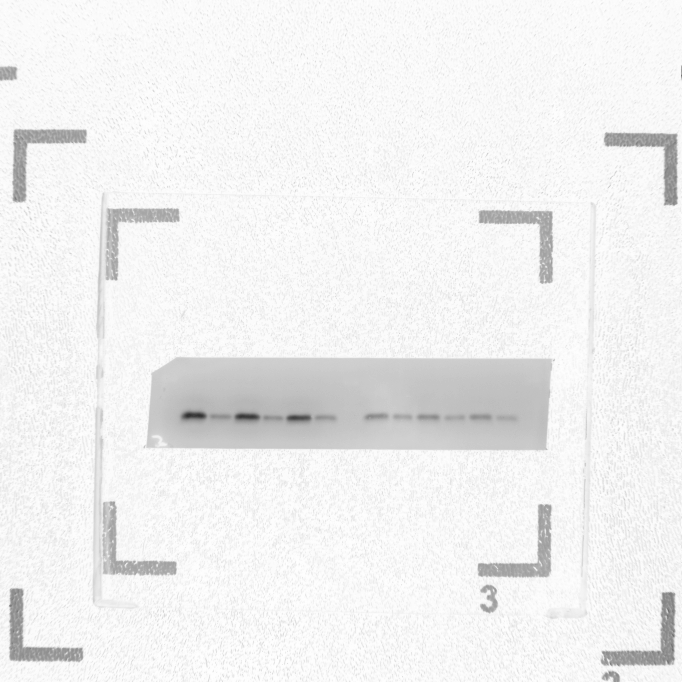

Supplement: Supplemental Information 3 [file peerj-11-14654-s003.zip › PSMD14-KD-marker -Figure 3B-3C.tif]

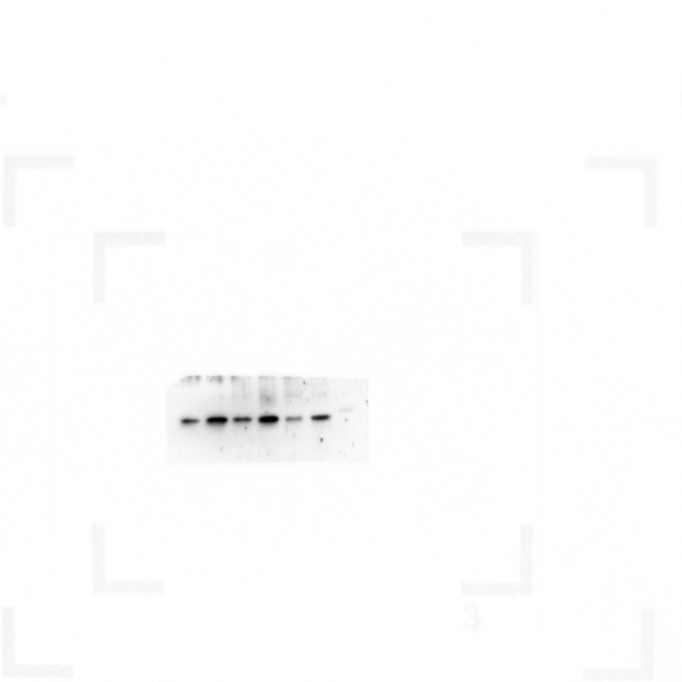

Supplement: Supplemental Information 3 [file peerj-11-14654-s003.zip › PSMD14-OE-Figure -3D.tif]

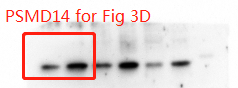

Supplement: Supplemental Information 3 [file peerj-11-14654-s003.zip › PSMD14-OE-Figure 3d-illustration.tif]

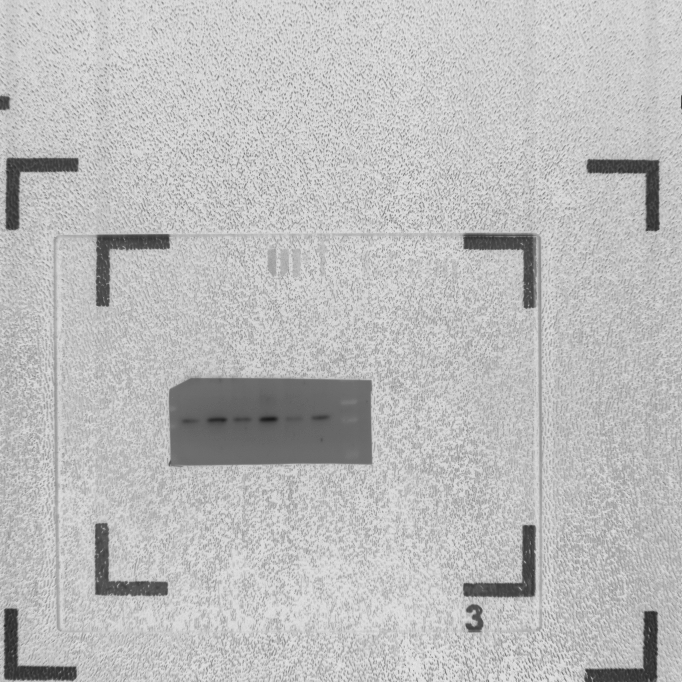

Supplement: Supplemental Information 3 [file peerj-11-14654-s003.zip › PSMD14-OE-marker-Figure -3D.tif]

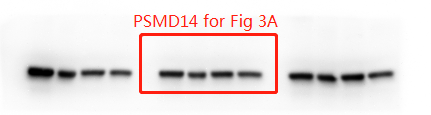

Supplement: Supplemental Information 3 [file peerj-11-14654-s003.zip › PSMD14-cell lines-Figure 3A-illustration.tif]

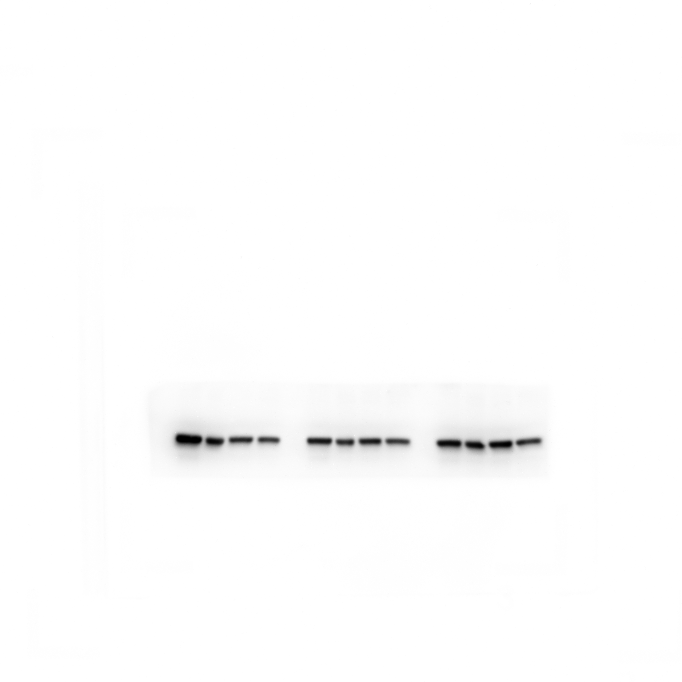

Supplement: Supplemental Information 3 [file peerj-11-14654-s003.zip › PSMD14-cell lines-Figure 3A.tif]

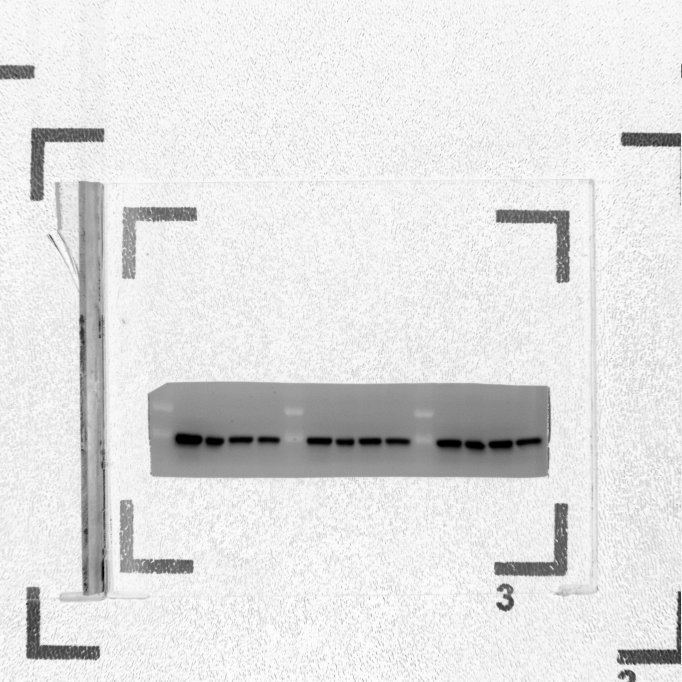

Supplement: Supplemental Information 3 [file peerj-11-14654-s003.zip › PSMD14-cell lines-marker -Figure 3A.tif]

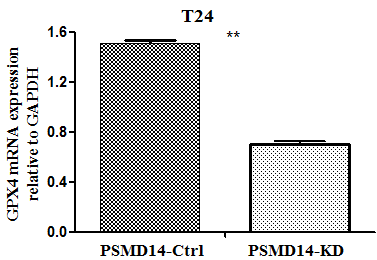

Supplement: Supplemental Information 3 [file peerj-11-14654-s003.zip › T24-E.png]

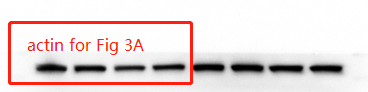

Supplement: Supplemental Information 3 [file peerj-11-14654-s003.zip › actin-Figure 3A-illustration.tif]

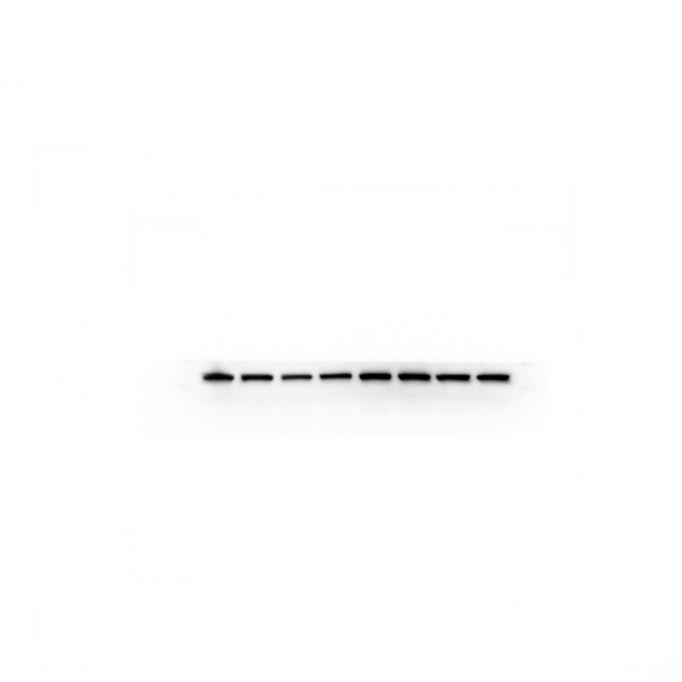

Supplement: Supplemental Information 3 [file peerj-11-14654-s003.zip › actin-Figure 3A.tif]

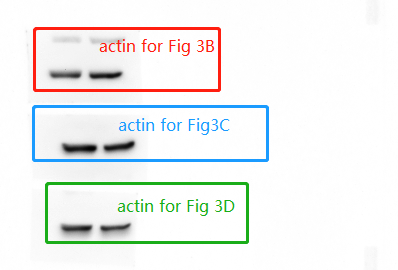

Supplement: Supplemental Information 3 [file peerj-11-14654-s003.zip › actin-Figure 3B-3D-illustration.tif]

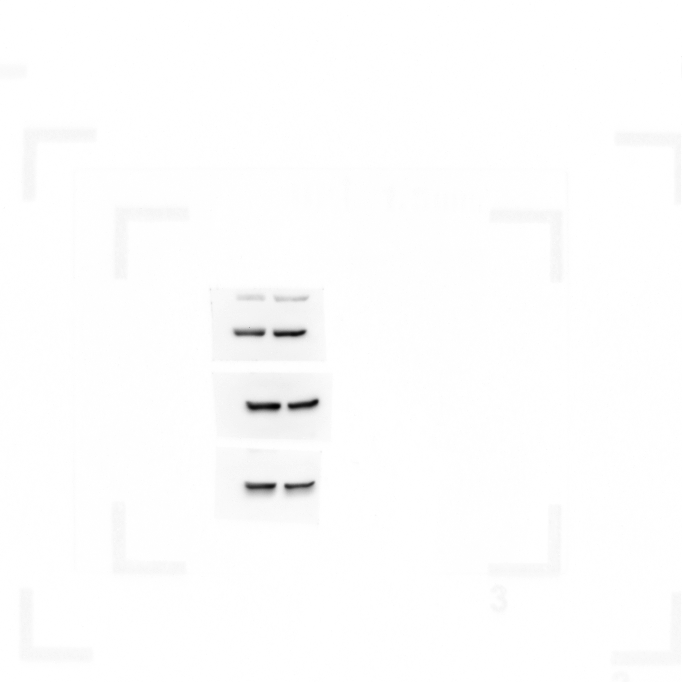

Supplement: Supplemental Information 3 [file peerj-11-14654-s003.zip › actin-Figure 3B-3D.tif]

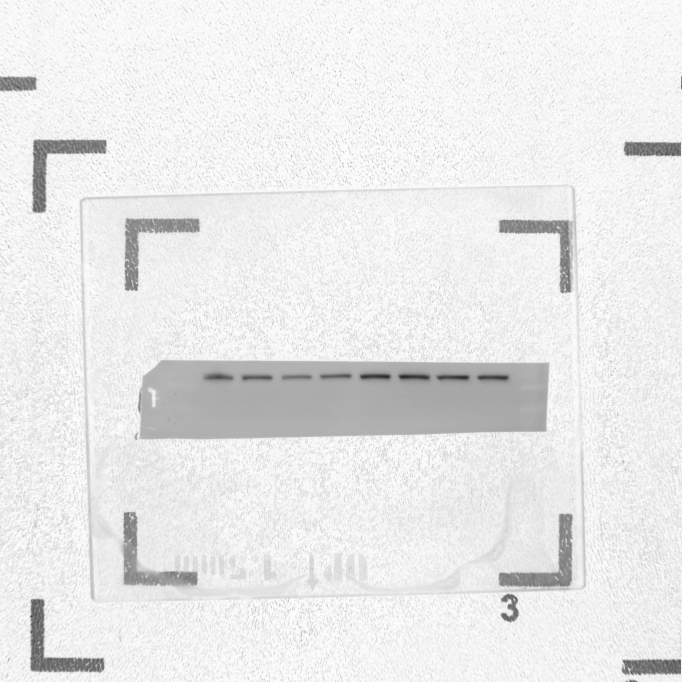

Supplement: Supplemental Information 3 [file peerj-11-14654-s003.zip › actin-marker -Figure 3A.tif]

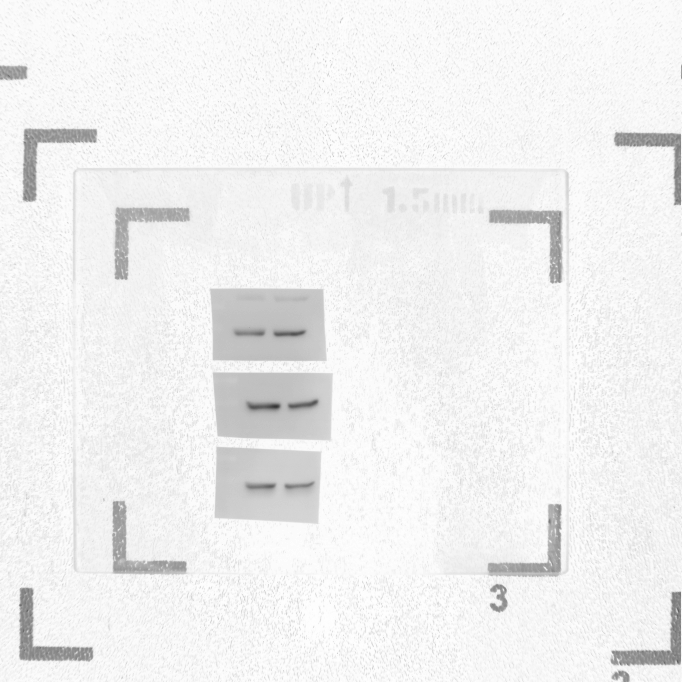

Supplement: Supplemental Information 3 [file peerj-11-14654-s003.zip › actin-marker -Figure 3B-3D.tif]

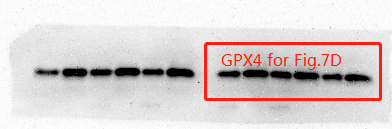

Supplement: Supplemental Information 4 [file peerj-11-14654-s004.zip › GPX4--Figure 7D-illustration.tif]

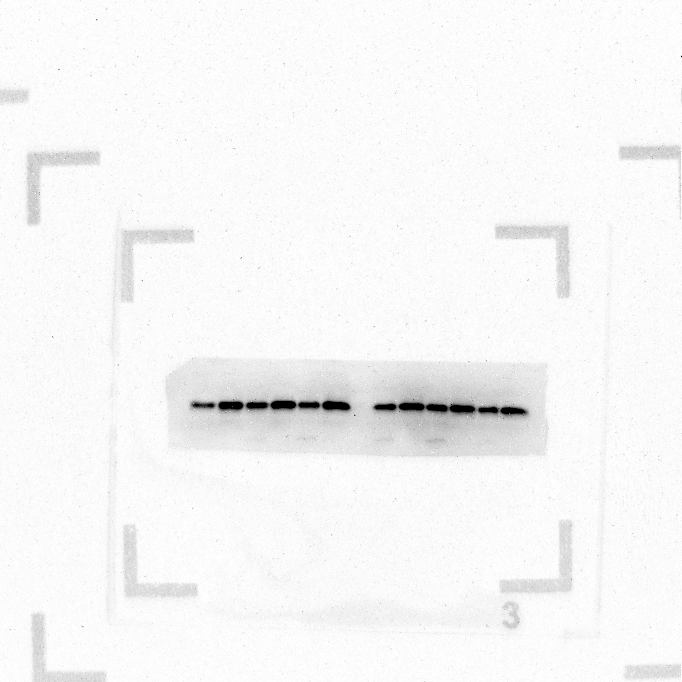

Supplement: Supplemental Information 4 [file peerj-11-14654-s004.zip › GPX4--Figure 7D-marker.tif]

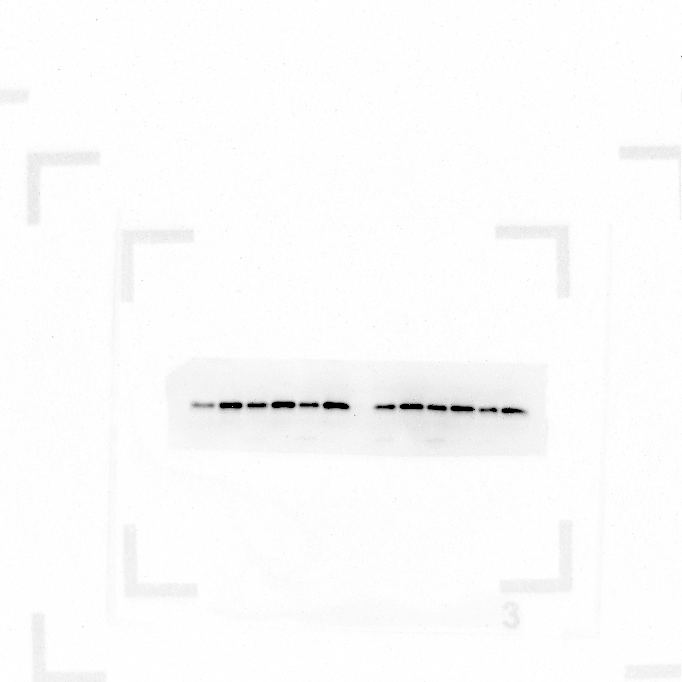

Supplement: Supplemental Information 4 [file peerj-11-14654-s004.zip › GPX4--Figure 7D.tif]

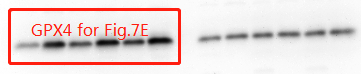

Supplement: Supplemental Information 4 [file peerj-11-14654-s004.zip › GPX4--Figure 7E-illustration.tif]

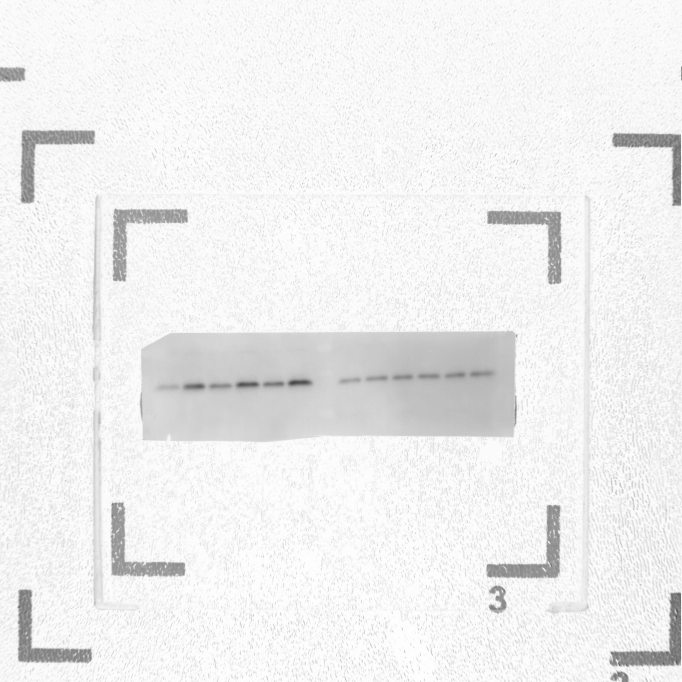

Supplement: Supplemental Information 4 [file peerj-11-14654-s004.zip › GPX4--Figure 7E-marker.tif]

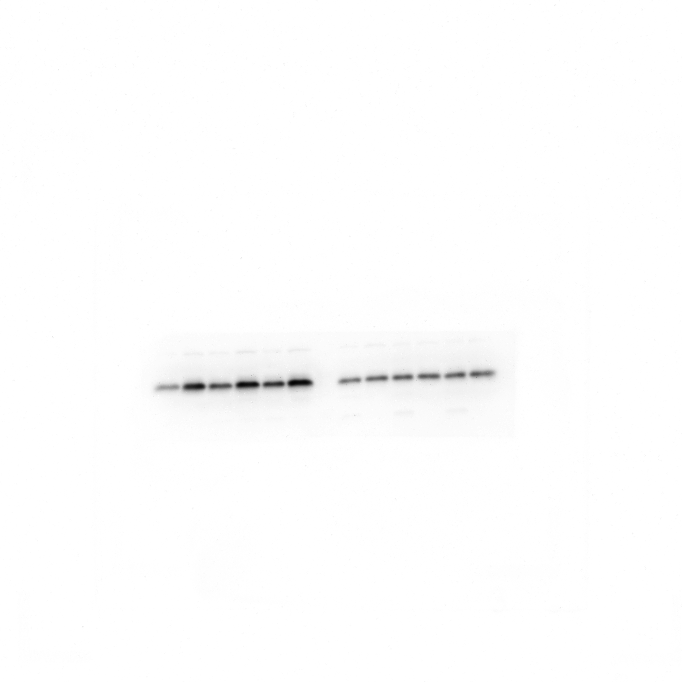

Supplement: Supplemental Information 4 [file peerj-11-14654-s004.zip › GPX4--Figure 7E.tif]

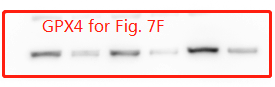

Supplement: Supplemental Information 4 [file peerj-11-14654-s004.zip › GPX4--Figure 7F-illustration.tif]

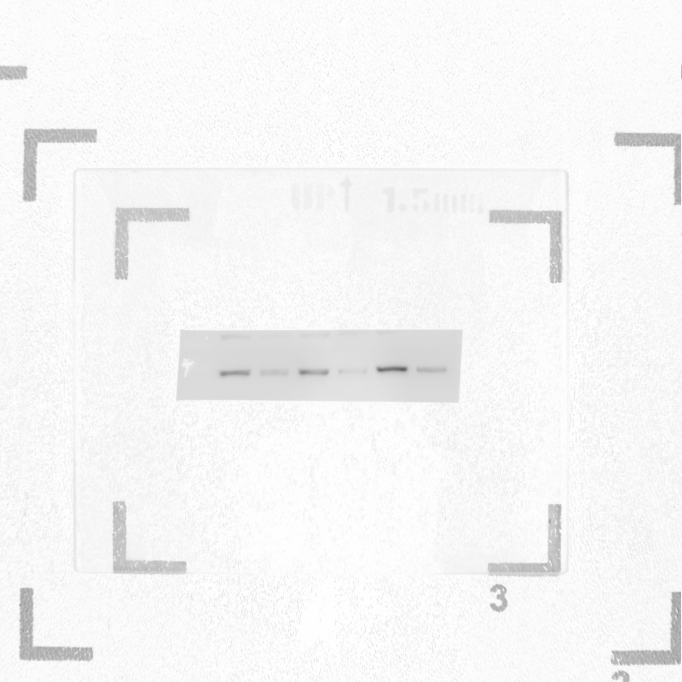

Supplement: Supplemental Information 4 [file peerj-11-14654-s004.zip › GPX4--Figure 7F-marker.tif]

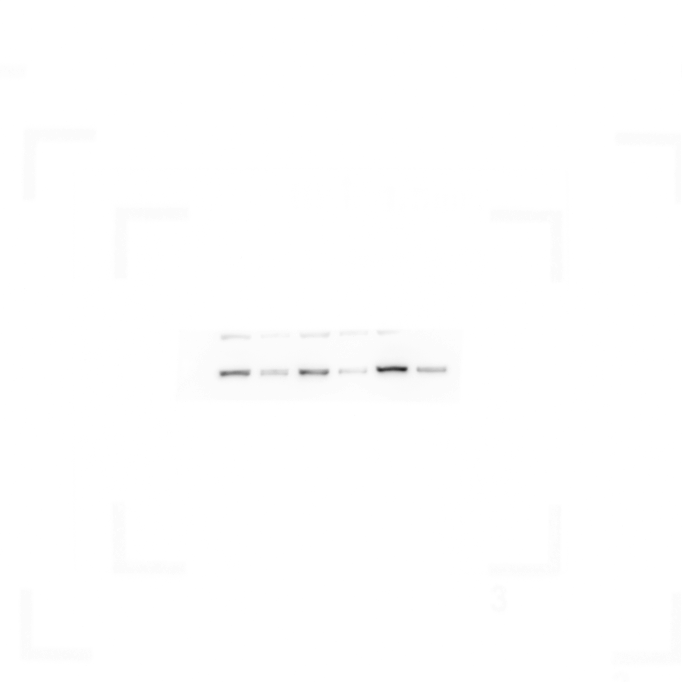

Supplement: Supplemental Information 4 [file peerj-11-14654-s004.zip › GPX4--Figure 7F.tif]

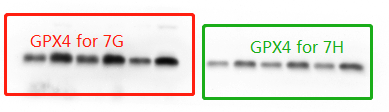

Supplement: Supplemental Information 4 [file peerj-11-14654-s004.zip › GPX4--Figure 7G-7H-illustration.tif]

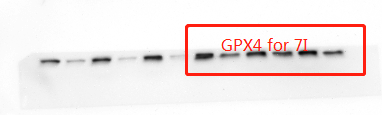

Supplement: Supplemental Information 4 [file peerj-11-14654-s004.zip › GPX4--Figure 7I-illustration.tif]

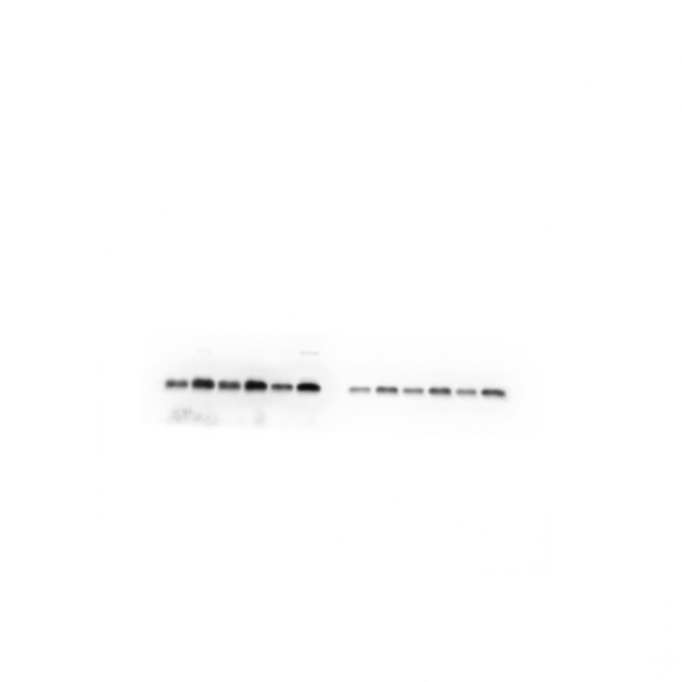

Supplement: Supplemental Information 4 [file peerj-11-14654-s004.zip › GPX4-Figure 7G - 7H.tif]

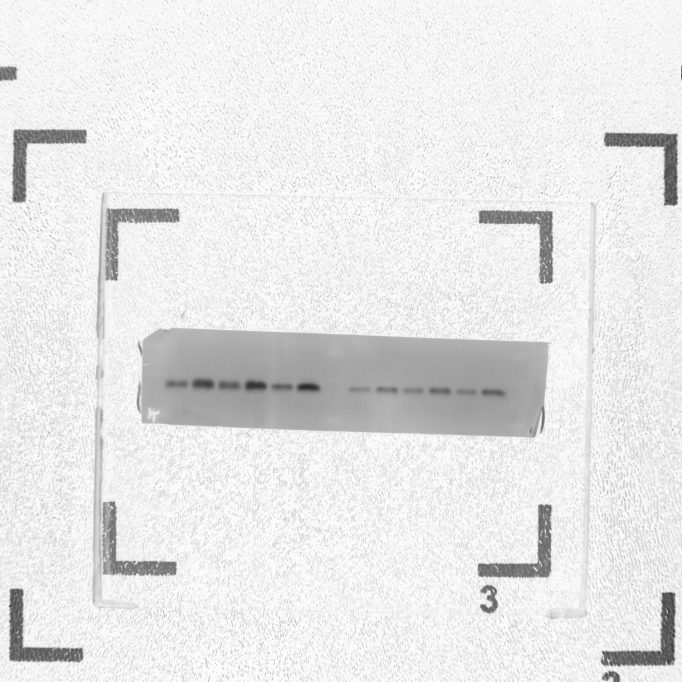

Supplement: Supplemental Information 4 [file peerj-11-14654-s004.zip › GPX4-Figure 7G -7H-marker.tif]

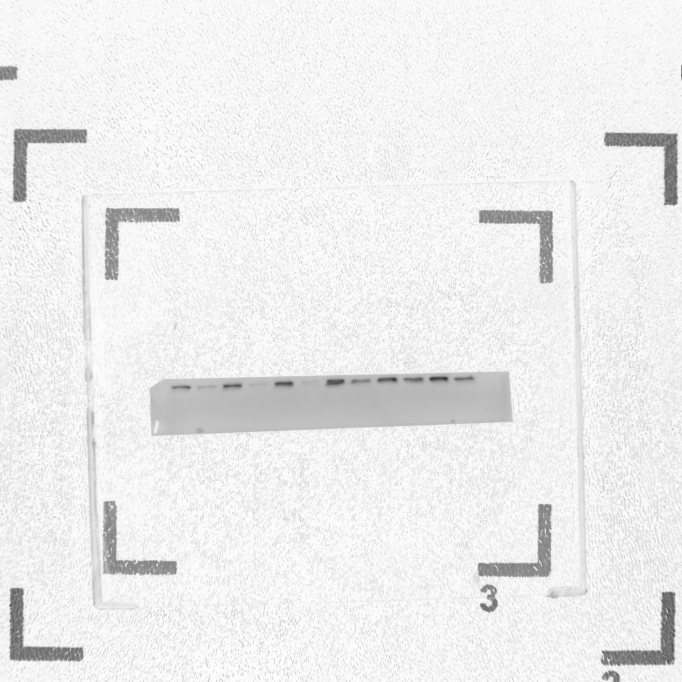

Supplement: Supplemental Information 4 [file peerj-11-14654-s004.zip › GPX4-Figure 7I-marker.tif]

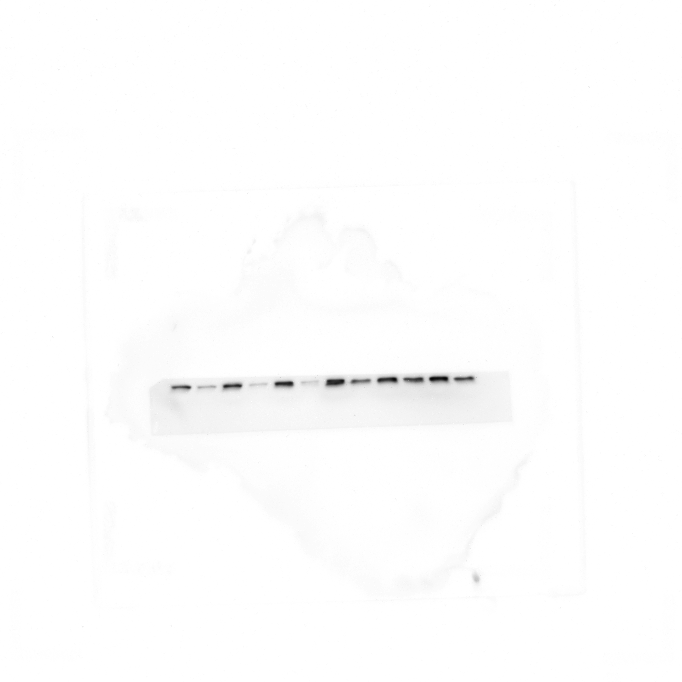

Supplement: Supplemental Information 4 [file peerj-11-14654-s004.zip › GPX4-Figure 7I.tif]

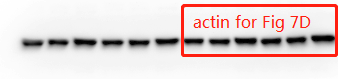

Supplement: Supplemental Information 4 [file peerj-11-14654-s004.zip › actin--Figure 7D-illustration.tif]

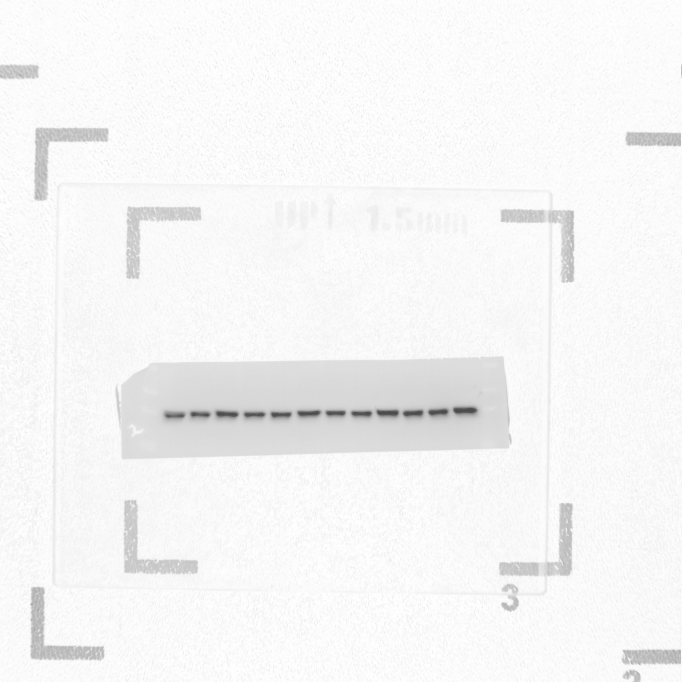

Supplement: Supplemental Information 4 [file peerj-11-14654-s004.zip › actin--Figure 7D-marker.tif]

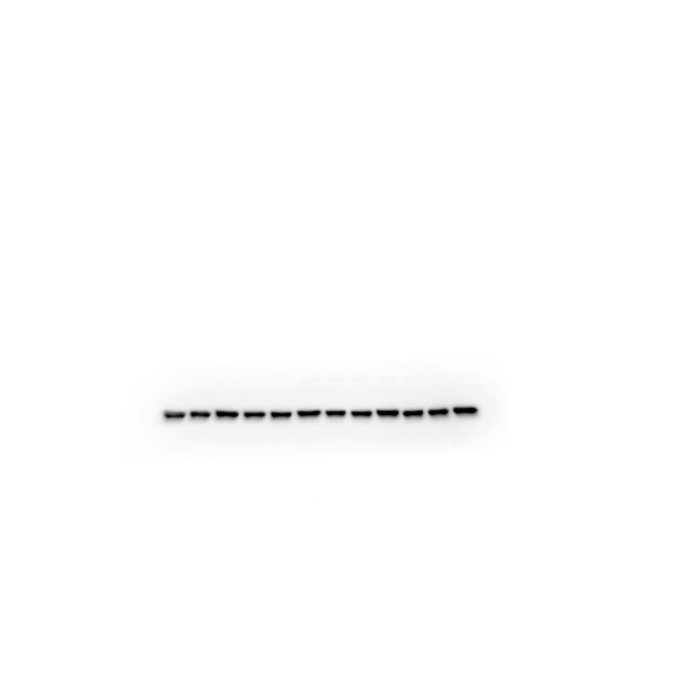

Supplement: Supplemental Information 4 [file peerj-11-14654-s004.zip › actin--Figure 7D.tif]

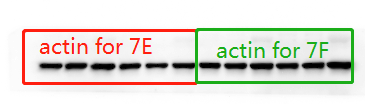

Supplement: Supplemental Information 4 [file peerj-11-14654-s004.zip › actin--Figure 7E-7F-illustration.tif]

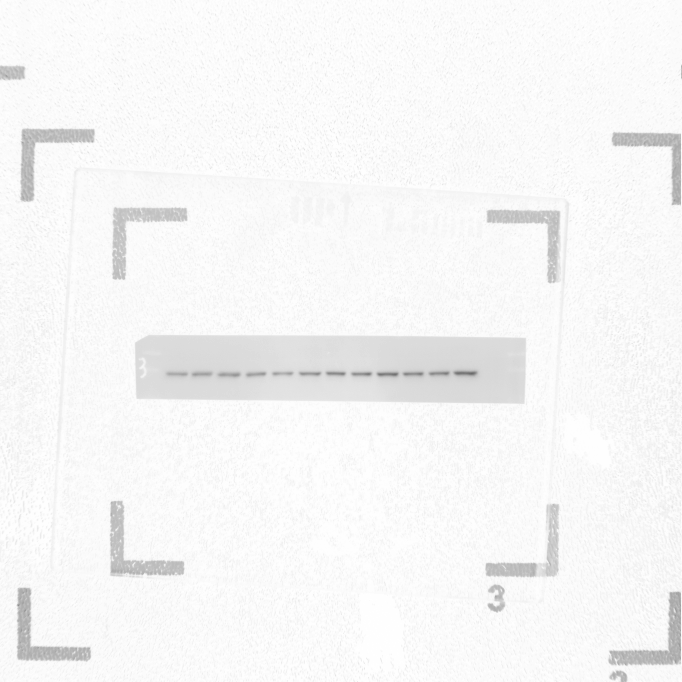

Supplement: Supplemental Information 4 [file peerj-11-14654-s004.zip › actin--Figure 7E-7F-marker.tif]

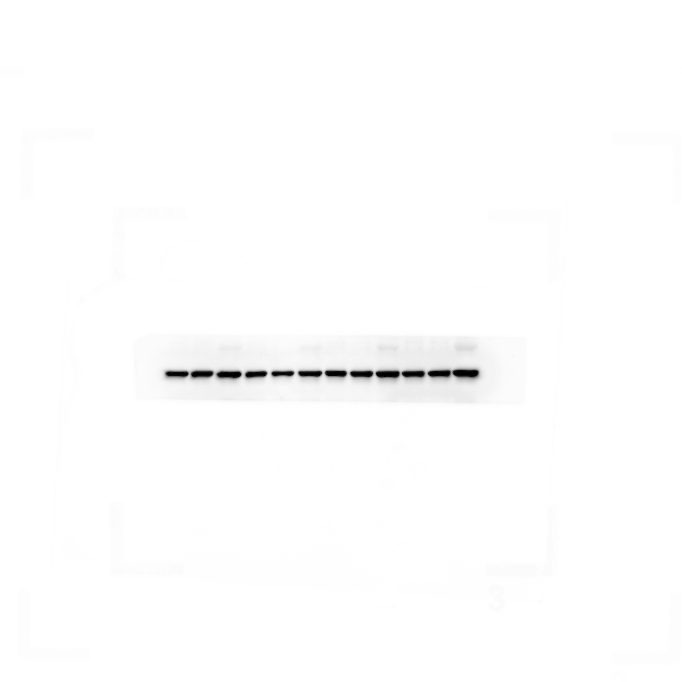

Supplement: Supplemental Information 4 [file peerj-11-14654-s004.zip › actin--Figure 7E-7F.tif]

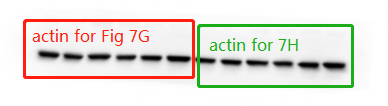

Supplement: Supplemental Information 4 [file peerj-11-14654-s004.zip › actin--Figure 7G-7H-illustration.tif]

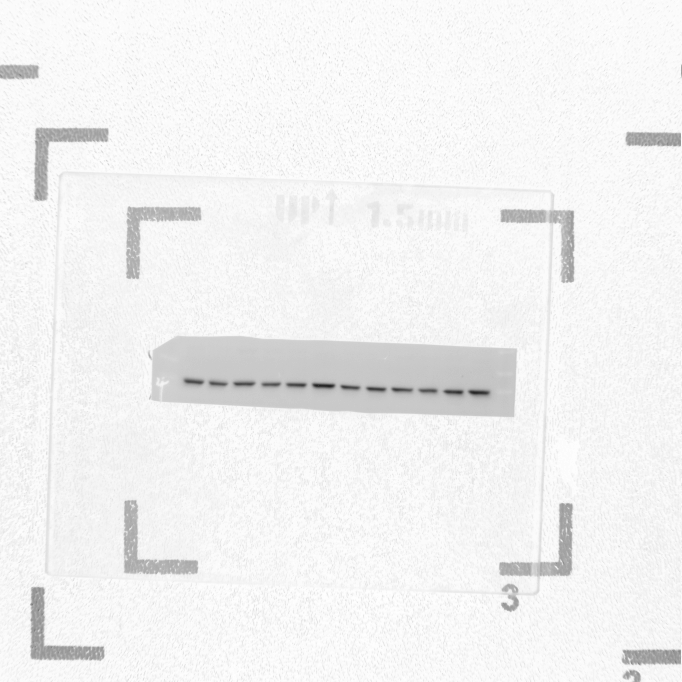

Supplement: Supplemental Information 4 [file peerj-11-14654-s004.zip › actin--Figure 7G-7H-marker.tif]

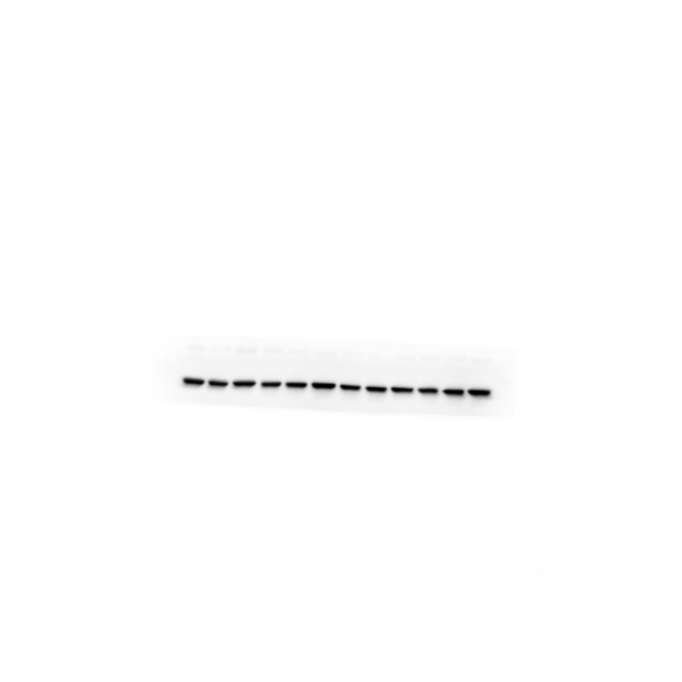

Supplement: Supplemental Information 4 [file peerj-11-14654-s004.zip › actin--Figure 7G-7H.tif]

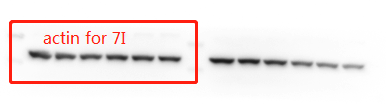

Supplement: Supplemental Information 4 [file peerj-11-14654-s004.zip › actin--Figure 7I-illustration.tif]

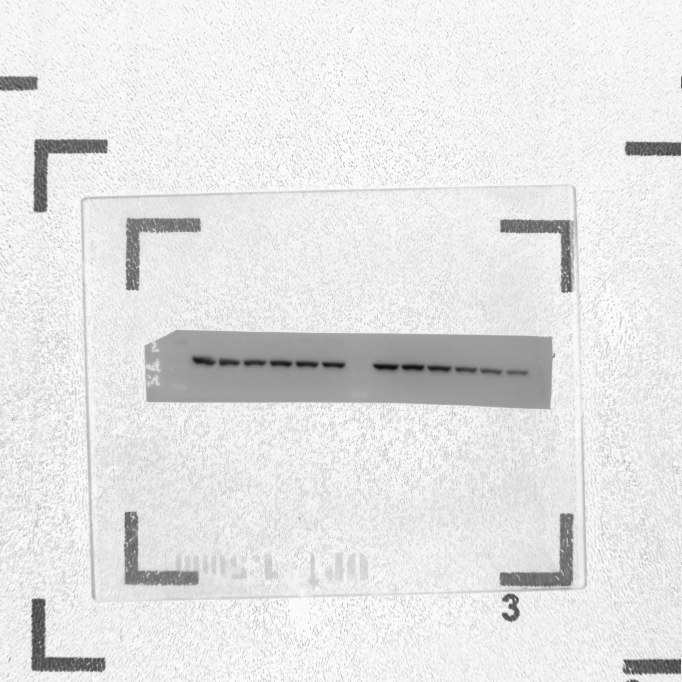

Supplement: Supplemental Information 4 [file peerj-11-14654-s004.zip › actin--Figure 7I-marker.tif]

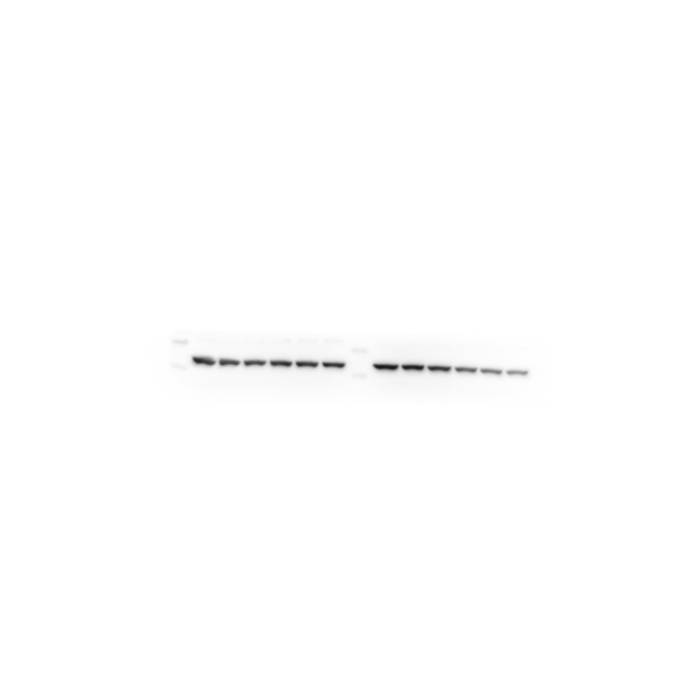

Supplement: Supplemental Information 4 [file peerj-11-14654-s004.zip › actin--Figure 7I.tif]
